# Supplementary material for: Widespread horse-based mobility arose around 2200 bce in Eurasia
Source: Nature. 2024 Jun 6;631(8022):819–25. doi: 10.1038/s41586-024-07597-5 (PMC11269178; doi:10.1038/s41586-024-07597-5)
Supplement: Supplementary file 1 — This Supplementary Information file contains the following sections: Section 1. Archaeological Contexts and Sample Information; Section 2. Radiocarbon Dating; Section 3. Genome Analyses; Section 4. Measuring temporal variations in the horse generation time; and references. [file 41586_2024_7597_MOESM1_ESM.pdf]

nature portfolio

https://doi.org/10.1038/s41586-024-07597-5

Supplementary information

Widespread horse-based mobility arose around 2200 BCE in Eurasia

In the format provided by the authors and unedited

Table of Contents

Section 1. Archaeological Contexts and Sample Information *4*

* Austria, Kittsee 4
* Austria: Roseldorf 4
* Britain: Alcester, context 1147 4
* Britain: Bradgate Park, Leicestershire 4
* Britain: Hungate, York 5
* China: Shanmava, Multilayered settlement 5
* Croatia: Šarengrad, graves nb. 26 and 52 5
* Croatia: Vinkovci, NaMa, grave nb. 3 5
* Croatia: Vinkovci, Makart 5
* Czechia: Bitozeves 6
* Denmark: Bredholm, Z.M.K. 2/1921 (Zoologisk Museum, København) 6
* Denmark: Føllenslev Mose, Z.M.K. 27/1947 (Zoologisk Museum, København) 6
* Denmark: Køge Å ved Spanager, Z.M.K. 8/1963 (Zoologisk Museum, København) 6
* Denmark: Ginnerup 6
* Denmark: Gørlev, Z.M.K. 104/1950 (Zoologisk Museum, København) 7
* Denmark: Hjortspring Kobbel, Z.M.K. 16/1922 (Zoologisk Museum, København) 7
* Denmark: Hovmarken, Z.M.K. 28.06.1862 (Zoologisk Museum, København) 7
* Denmark: Nytorv, Z.M.K. unregistered (Zoologisk Museum, København) 7
* Denmark: Tuse Skole, Z.M.K. 25/1995, MHO 315/89 (Zoologisk Museum, København) 7
* Denmark: Vejen, Z.M.K. 12.07.1900 (Zoologisk Museum, København) 7
* Denmark: Rislev, Z.M.K. 114/1945 (Zoologisk Museum, København) 8
* Denmark: Langhøj, Z.M.K.19/2012, TAK 1518 (Zoologisk Museum, København) 8
* Denmark: Ulvehøj, Z.M.K. 118/1971 (Zoologisk Museum, København) 8
* France: École Nationale Vétérinaire de Maison Alfort (ENVA, Val-de-Marne 94) 8
* France: Le Cendre - Gondole - Les piots 8
* France: Orcet, La Roche Blanche - L'Enfer, Department of Puy-de-Dôme 8
* France: Pech Maho 9
* Germany: Krefeld-Gellep 9
* Germany: Rathewitz 9
* Hungary: Bakonszeg-Kádárdomb 9
* Hungary: Berettyóújfalu-Szilhalom 10
* Hungary: Budapest-Királyok Útja 293 10
* Hungary: Dunakeszi-Székesdűlő 10

* Hungary: Gáborján-Csapszékpart 10
* Hungary: Százhalombatta-Földvár 11
* Iran: Dava Göz 11
* Israel: Sepphoris 12
* Italy: Tarquinia 12
* Kazakhstan: Karatomar 12
* Moldova (or Moldavia): Odaia 12
* Mongolia: Biluut-2 (Khuiten Gol Delta), Pazyryk Burials 2-1 and 2-2 13
* Mongolia: Egyin Gol 13
* Mongolia: Tamir, tomb nb. 13 and 22 13
* Mongolia: Burgast, Bayan Ölgii province 13
* Mongolia: Zoolongiin am 1-1, catacomb №2 13
* Poland: Tominy 14
* Russia: Krasny Gorodok settlement, Abashevo 14
* Russia: Arzhan-2, burial nb. 5 14
* Russia: At Daban 13 14
* Russia: Brusyany IV 15
* Russia: Chekon 15
* Russia: Derkul 15
* Russia: Filippovka II, kurgan 1, grave nb. 2 15
* Russia: Idzhil-I, kurgan 1, Kalmykia 15
* Russia: Industryia 15
* Russia: Ipatovo 3, Kurgan 2, Animal Complex 13 16
* Russia: Katanda-II, The "Big Katandinsky" Kurgan 16
* Russia: Khankarinsky Dol (Mound group), Mound No. 11 16
* Russia: Krasnosamarkskoe IV, kurgan and settlement 17
* Russia: Sadgorod IV, kurgan 2, sacrificial complex 2 17
* Russia: Shumaevo I, kurgan5 17
* Russia: Tyubyak 17
* Russia: Yaloman-II, mound No. 46a 17
* Russia: Zayukovo-3 burial ground, sacrificial pits 7 and 8 18
* Slovenia: Bled, Pristava, grave nb. 18 18
* Spain: Alorda Park, Tarragona 18
* Spain: Can Roqueta-Torre Romeu, Sabadell, Barcelona 18
* Spain: El Graell, Vic, Osona, structure E-42 18

*** Spain: Hereuet, Lleida** **19**

*** Spain: Puig de Sant Andreu, Girona** **19**

*** Sweden: Eketorp, Gräsgård parish, Öland** **19**

*** Sweden: Skedemosse, Gärdslösa parish, Öland** **19**

*** Tajikistan: Shohidon, grave №20** **19**

*** Türkiye: Çadir Hoyük** **20**

*** Türkiye: Yenikapı, Istanbul** **20**

*** Ukraine, Husatyn** **20**

Section 2. Radiocarbon Dating ***21***

Section 3. Genome Analyses ***22***

Comparative panels **22**

Sequence data trimming, rescaling, and pseudo-haploidization **23**

Relative error rates **23**

The genetic makeup of CWC and Central European horses **24**

Population structure **26**

Horse inbreeding levels **26**

Reconstruction of demographic trajectories **27**

Section 4. Measuring temporal variations in the horse generation time ***29***

Motivation **29**

Mutation clock **29**

Recombination clock **30**

Validation using coalescent simulations **32**

Filtering out horse specimens unsuitable for regression analyses **32**

Temporal shifts in generation times **33**

Supplementary Reference List ***36***

Supplementary Information

Section 1. Archaeological Contexts and Sample Information

The following section provides a short summary of the new archaeological contexts associated with the specimens sequenced in this study, sorted by country of origin. For previously-reported horse genomes, the description of their archaeological contexts can be found in the original publications (*eg.* Aleksandrovskoe IV, Bestamak, Bol'shekaraganskii, Halvai, Kamennyi Ambar 5, Kozhai, Nikolskaya, Noviye Kluchi III, Ouren', Pershinskaya, Potapovka, Repin Khutor, Serpievskaya, Sosnovka 1, Utevka VI, Uvarovka II, and Verkhnegubakhinskaya were fully described by Librado and colleagues (2021)[2]). The archaeological context related to the donkey sample Albufeira1x2_Spa_1224, from Albufeira, Portugal, was described by Todd and colleagues (2022)[59]. The name of each sample concatenates the original name of the material analysed, its country of origin, and the midpoint of its Common Era (CE) age, or appended with an 'm' suffix if Before Common Era (BCE). The different fields defining sample names are separated by '_' symbols.

*** Austria, Kittsee**

[48.101469, 17.04521]

Three remains of horse (one tooth, 1 us ungulare, 1os coronale) were discovered at the Kittsee site, during a rescue excavation in 1995. These bones stem from a settlement pit of the Late Neolithic Baden Culture together with fragments of a clay model of a carriage, an artefact characteristic for this culture. Typology of finds and the radiocarbon date obtained for the sample presented in this study, KT46_Aus_m3240 (UCIAMS278407, 4,560±20 uncal. BP, i.e. 3,371-3,109 cal. BCE; Table S1), places the settlement of Kittsee around the transition of the older phase of the Baden Culture (Boleráz) to its younger one (Ossarn).

*** Austria: Roseldorf**

[48.645861, 15.929631]

Two horse remains (SRNHM004_Aus_m294 and SRNHM007_Aus_m301) were sampled from the bone assemblage of a La Tène settlement in Roseldorf, Lower Austria. The tooth was discovered in course of the excavation of the sanctuary (object 14)[69]. Numerous horse bones were found in a context associated with food disposal (cf. object 1)[69]. Therefore, a comparable context could possibly be assumed for object 14. Radiocarbon dating indicated that both samples died during the 4th and 3rd centuries BCE (UCIAMS250230, 2,235±20 uncal. BP, OxCal20 382-206 cal. BCE, and; UCIAMS250231, 2,260±20 uncal. BP, OxCal20 394-208 cal. BCE, respectively; Table S1).

*** Britain: Alcester, context 1147**

[52.211, -1.8676]

The specimen sampled for DNA analysis and radiocarbon dating (Alcester1147_UK_169) was recovered during excavations by Archaeological Investigation Ltd at the site of Bleachfield Street, Alcester, Warwickshire, during year 2005. The site is located at the south-west corner of Alcester, at some distance from the River Arrow. The horse specimen consists of a pars petrosa of the temporal bone of the skull, and was recovered from archaeological context 1147. This context belongs to the earliest phase at the site (Phase 1), assigned by the excavators to the early Roman period, *ie.* late 1st - early 2nd century CE, and confirmed through radiocarbon carbon dating (UCIAMS199290, 1,880±15 uncal. BP, OxCal20 122-215 cal. CE; Table S1). The pit itself, 1.7 meter long and 1.7 meter wide, and 0.15 meter depth, was filled with dark brown silty loam. Besides the skull in question, the pit contained fragments of a right and a left mandible - presumably belonging to the same individual – and a horse tibia, as well as a small number of cattle bone fragments.

*** Britain: Bradgate Park, Leicestershire**

[52.69, -1.22]

The horse bone assemblage from Bradgate Park, Leicestershire, derived from the fill of a raised platform, located just beyond the north wall of a substantial aristocratic stable block[70]. Analysis of associated ceramics

yielded consistent dates spanning 1680-1750 CE. Radiocarbon dating of the petrous bone of a horse skull (BGPSK1_UK_1661) returned an uncalibrated date of 1,527-1,795 cal. CE (OxCal20, UCIAMS250206, 260±20 uncal. BP; Table S1). Taken together, the bone assemblage likely dates to the late 17th-early 18th centuries CE.

*** Britain: Hungate, York**
[53.9592235, -1.0779786]
The site of Hungate, York, was excavated between 2006 and 2011 by York Archaeological Trust. It represents a small Roman period cemetery, overlaid by extensive medieval and later activity, much of which corresponds to garbage deposition on 'waste' ground. Sample VEM107_UK_956 was from fills of a pit in Area H2, of Anglo-Scandinavian phase associated with timber buildings. Stratigraphic and dating integrity are secure. This context pre-dates, and is separate from, the substantial re-deposition that occurs later in the Hungate sequence. Radiocarbon dating indicated that the specimen died between 895 and 1,016 CE (UCIAMS182877, 1,085±15 uncal. BP; Table S1).

*** China: Shanmava, Multilayered settlement**
37°00'38.6"N 106°22'57.7"E [37.010722, 106.382694444444]
A multi-layered complex of archaeological sites belonging to different time periods is located in the river valley near the village of Shanmava, near the city of Guan (Ningxia Hui Autonomous Region, China). The section of the river bank revealed a cultural layer of a settlement, which may correspond to an ancient city, dating to the end of the Zhanguo period. The Zhanguo period, at the beginning of the Han period, is the heyday of ancient Chinese culture, the time of its expansion and the realization of the idea of uniting the destinies of China. The horse sample Shanx1_Chi_m112 was collected by A. Tishkin when visiting the site in 2016, and was radiocarbon dated to 174-49 BCE (OxCal20 calibrated years) (UCIAMS229412, 2,105±20 uncal. BP; Table S1).

*** Croatia: Šarengrad, graves nb. 26 and 52**
[45.23145, 19.280436]
Horse skeletons and bones were discovered in the early Medieval cemetery of Šarengrad-Klopare, situated on a steep slope above the Danube river during a rescue excavation in 2016 and 2017[71,72]. Based on stratigraphy and the nature of the assemblage found, the site was dated to the 8th century CE, which corresponds to the Late Avar Period (2nd Khaganate). For Croatia, the Late Avar cemetery of Šarengrad represents one of the prominent sites in the Danube region and southern Pannonia. The samples were taken from grave nb. 26 (tooth, SRKRO002_Cro_722)[72], and grave nb. 52 (petrosal bone, SRKRO001_Cro_331). Radiocarbon dating results were consistent with the described archaeological context (UCIAMS250238, 1,285±20 uncal. BP, OxCal20 670-774 cal. CE, and; UCIAMS250237, 1,295±20 uncal. BP, OxCal20 664-775 cal. CE, respectively; Table S1).

*** Croatia: Vinkovci, NaMa, grave nb. 3**
[45.288753, 18.8008]
The town of Vinkovci in north-eastern Croatia delivered a burial site from the late Hallstatt period, which was discovered during archaeological rescue excavations[73]. At the location of the NaMa department store, the graves were excavated in poor weather conditions in the winter of 1976/77, and were, therefore, insufficiently documented. These are skeleton tombs, which are from the 2nd half of the 5th and 1st half of the 4th century BCE, with typical south Pannonian equipment, such as costume, weapons and ceramics[73]. Three horse burials were also discovered in the area of the burial ground, two of them with bridles[73]. The sample collected for DNA analyses belongs to grave nb. 3 (SRKRO009_Cro_m453), which was placed between human graves without equipment. Radiocarbon dating indicated that the specimen most likely died during the 5th century BCE (UCIAMS250225, 2,365±20 uncal. BP, 514-391 cal. BCE; Table S1).

*** Croatia: Vinkovci, Makart**
[45.286211, 18.800061]

A Roman necropolis was discovered in the north-eastern Croatian town Vinkovci (Colonia Aurelia Cibalae), between the park Lenija and the river Bosut. It is believed to date to the 3rd and the 5th centuries CE (unpublished report for the City museum Vinkovci, Archaeological Department by A. Rapan Papeša 2010). One petrosal bone from the horse skull that was placed underneath the right shoulder of the human body, and was sampled for DNA analysis and radiocarbon dating (SRKRO011_Cro_331). The latter was dated twice, and returned consistent dates between the 3rd and the 5th centuries CE (UCIAMS250226, 1,735±20 uncal. BP, OxCal20 249-402 cal. CE, and; UCIAMS251144, 1695±20 uncal BP, OxCal20 259-415 cal. CE; Table S1).

*** Czechia: Bitozeves**
[50.3662084, 13.65182266]
The material selected for ancient DNA analysis was taken from the archaeological find of partial skeletons of two horse individuals found together with two human skulls in feature No. 5/2017 at Bitozeves (distr. Louny, Czechia). The archaeological excavation at Bitozeves was conducted by ÚAPPSZČ - Most under supervision of Věra Sušická in 2017. The archaeological situation and osteological analysis are not published yet, but a preliminary report places the find to the Migration Period. Sample PRA29_Cze_471 comes from the petrosum of the horse No. 1, which is a 13-16 years-old male, according to the morphological features. The sex assignment was confirmed by DNA analysis, and radiocarbon dating indicated that the specimen died during the 5th-6th centuries CE (UCIAMS208889, 1,630±15 uncal. BP, OxCal20 407-535 cal. CE; Table S1).

*** Denmark: Bredholm, Z.M.K. 2/1921** (Zoologisk Museum, København)
[55.067434, 10.607282]
Individuals P128_Den_1458, P129_Den_1457, P130_Den_1459 and P131_Den_1461 were sampled from loose petrosal bones, excavated from an Iron Age settlement on Bredholm (Stryno parish), a small island located in the South Funen archipelago, Denmark (Fynske Minder: Odense Bys Museers Årbog 2015 Forlaget Odense Bys Museer, pers. Comm.). The site consisted of a ~8-9 meter-large ring ditch, where bones from at least 14 horses were identified, together with an iron knife, attributed to the Late Iron Age. All four specimens analysed in this study were radiocarbon dated and were found to be intrusive material from the 15th century CE (UCIAMS199276, UCIAMS199296, UCIAMS199303, UCIAMS199304; Table S1).

*** Denmark: Føllenslev Mose, Z.M.K. 27/1947** (Zoologisk Museum, København)
[55.714655, 11.717578]
Føllenslev Mose corresponds to a possible sacrificial site, located in West Zealand, Denmark, dating back to the Iron Age, and where large quantities of wood, together with a flint axe and bones of humans and domesticated animals, including individual P133_Den_O1663, were excavated[74]. This specimen was, however, identified as intrusive through radiocarbon dating, which assigned the specimen to the modern period (UCIAMS199614, 185±15 uncal. BP, OxCal20 >1,663 cal. CE; Table S1).

*** Denmark: Køge Å ved Spanager, Z.M.K. 8/1963** (Zoologisk Museum, København)
[55.67594, 12.56553]
Sample P134_Den_862 was unearthed by the Køge å, a small river located next to Spanager, East Zealand, Denmark. This specimen was believed to be from the Middle Ages, which was confirmed through radiocarbon dating (UCIAMS199249, 1,165±15 uncal. BP, OxCal20 772-951 cal. CE; Table S1).

*** Denmark: Ginnerup**
[56.41134, 10.74481]
Ginnerup is located on the Djursland peninsula in eastern Jutland, Denmark. At the time of occupation, it was a coastal site on a promontory, bordering the c. 1 kilometer-wide Kolindsund sound located to its south and a narrow branch of the sound on its eastern side. The surrounding, undulating landscape to the north, east and west is made up of sandy moraine and meltwater plateaus between 15 and 60 m.a.s.l. Samples GIN1020_Den_m3000 and GIN1055_Den_m3000 are from horse remains excavated in layer 7 in structure A4, a natural depression filled with an undisturbed sequence of archaeological layers[75]. Layer 7 has been dated to 3000-2920 BC by modelling of 11 ^{14}C-dates from a stratigraphy comprising four layers (see Klassen et al.

(2023)[75], pages 47-49, where individual dates and details on sample treatment and modelling statistics are given). The archaeological content of this layer derives from the later part of a transitional phase between the Neolithic Funnel Beaker (TRB) and Pitted Ware (PWC) cultures, comprising TRB pottery decorated in MN A II/III and Ferslev style and PWC pottery. Samples GIN396_Den_m3000, GIN489_Den_m3000 and GIN561_Den_m3000 all were obtained from horse remains from context K68 in structure A1 (ditch with numerous recuttings, unpublished, not yet dated by ^{14}C). K68 represents a deposition of shells containing TRB (MN A II) and PWC pottery. A slightly higher percentage of TRB vs. PWC elements of the pottery from K68 compared to that from layer 7 in structure A4 indicates a slightly older date[75,76].

*** Denmark: Gørlev, Z.M.K. 104/1950** (Zoologisk Museum, København)
[55.533331, 11.233332]
Excavation of a basement in the village of Ulstrup, near Gørlev, western Zealand, provided an animal bone assemblage from various domestic mammals including the domestic cat, as well as one loose petrous bone from a horse, P187_Den_1334. The bones are thought to be from the Danish Middle Ages or later. Radiocarbon dating of the specimen confirmed its medieval association (UCIAMS199294, 670±15 uncal. BP, OxCal20 1,281-1,386 cal. CE; Table S1).

*** Denmark: Hjortspring Kobbel, Z.M.K. 16/1922** (Zoologisk Museum, København)
[54.9637743, 9.5865551]
Hjortspring Kobbel is a pre-Roman Iron Age weapons hoard from south Jutland, where skeletal elements from individual P137_Den_m291 were unearthed. Radiocarbon dating confirmed the association of this specimen with the Iron Age (UCIAMS199274, 2,230±15 uncal. BP, OxCal20 376-205 BCE; Table S1).

*** Denmark: Hovmarken, Z.M.K. 28.06.1862** (Zoologisk Museum, København)
[54.965466, 12.391368]
Sample P138_Den_1224 was unearthed from Hovmarken, an area located north of the Copenhagen city, in what is today Gentofte, Denmark. The bones were collected by Japetus Steenstrup, but no archaeological excavation has been conducted at this site. The finds were thought to date back to the Iron Age; however, the horse sample analyzed returned a medieval date (UCIAMS199275, 825±15 uncal. BP, OxCal20 1,180-1,267 cal CE; Table S1).

*** Denmark: Nytorv, Z.M.K. unregistered** (Zoologisk Museum, København)
[55.47736, 9.13794]
Sample P139_Den_1381 was unearthed at Nytorv, in the centre of the Copenhagen city, Denmark and was presumed to date back to the Danish Middle Ages. Radiocarbon dating, however, indicated that the specimen belonged to the medieval period (UCIAMS199628, 535±20 uncal. BP, OxCal20 1,327-1,434 cal. CE; Table S1).

*** Denmark: Tuse Skole, Z.M.K. 25/1995, MHO 315/89** (Zoologisk Museum, København)
[55.7126691, 11.6300396]
The complete skeleton of a foal was found in a pit unearthed during sewerage work in Holbaek, Sjæland, without any precise archaeological context, except that it was found during the demolition of a main sewage line between Holbæk and Hagested (Iron Age to Medieval; Arkæologiske udgravninger i Danmark, Katalog 1989). This individual, P135_Den_1806, was radiocarbon dated to the modern period (UCIAMS199273, 120±15 uncal. BP, 1,687-1,925 cal. CE; Table S1).

*** Denmark: Vejen, Z.M.K. 12.07.1900** (Zoologisk Museum, København)
[55.47736, 9.13794]
Vejen is a small creek in central Jutland, near the town of Vejen, Denmark, which yielded a large number of burnt and unburnt bones of domesticated animals, including pigs and cows, and to a lesser extent, horses. These include sample P140_Den_O1660, which was radiocarbon dated to the modern time period (UCIAMS199627, 185±20 uncal. BP, OxCal20 >1,660 cal. BCE; Table S1).

*** Denmark: Rislev, Z.M.K. 114/1945** (Zoologisk Museum, København)
[55.2817532, 11.7238352]
Rislev is an Iron Age sacrificial site, located in South Zealand, Denmark, which consists of some artifacts, a large number of bones of domesticated animals, including the skull of individual P188_Den_149. The site also plays host to the complete skeletons of two humans and various additional human bones[77]. A total of 11 horses, including old, young, male and female animals, were identified on site, from bones of the head, feet and tail, which are thought to have been deposited there as offerings. Radiocarbon dating indicated that the individual analyzed genetically died between 83 and 215 cal. CE (OxCal20, UCIAMS199292, 1,890±20 uncal. BP; Table S1).

*** Denmark: Langhøj, Z.M.K.19/2012, TAK 1518** (Zoologisk Museum, København)
[55.013447, 9.7766747]
The headless remains of a cow and the skull of a horse, individual P189_Den_1541, were found together in a ditch during an excavation near Langhøj, Hvidovre, west of Copenhagen, Denmark. They were thought to have been dumped in the ditch at the same time. However, the cow remains were radiocarbon dated to 253±30 uncal. BP. The horse was found to be slightly older (UCIAMS199302, 370±15 uncal. BP, OxCal20, 1,457-1,624 cal. CE; Table S1).

*** Denmark: Ulvehøj, Z.M.K. 118/1971** (Zoologisk Museum, København)
[56.032262, 8.7141755]
The skull of one horse, referred to as individual P191_Den_O1663 in this study, was found by a local teacher at the site of a previous Bronze Age burial mound known as Ulvehøj, outside Gundsømagle, Zealand. Radiocarbon dating of the specimen indicated, however, that it was intrusive and dated to the modern period (UCIAMS199291, 185±15 uncal. BP, OxCal20 >1,663 CE; Table S1).

*** France: École Nationale Vétérinaire de Maison Alfort (ENVA, Val-de-Marne 94)**
The DNA was extracted from a tooth collected on the skeleton of an old mare over 15 years-old. The skeleton entered the collections of the Maison Alfort veterinarian school in 1903, and was recorded as "*Squelette de jument irlandaise*" *ie.* the skeleton of an Irish mare (piece #111, located window 23). It is still curated as part of the Fragonard Museum. The museum book records indicate that the skeleton has been artificially set-up by Dr Petitcolin in 1903, with skeletal pieces that may have been collected earlier, between 1882 and 1903. Radiocarbon dating returned estimates that were consistent with the museum records (Alfort3_Fra_1806; UCIAMS199266, 120±15 uncal. BP, OxCal20 1,687-1,925 cal. CE; Table S1).

*** France: Le Cendre - Gondole - Les piots**
[45.7205658, 3.1856364]
The site of Gondole « les Piôts » was excavated by U. Cabezuelo and his team (Inrap) to the side of a gallic oppidum. A pit (F137) dated from the 2nd century BCE delivered a set of eight men and eight horses associated in the grave. Humans and equids are all males, lying on their side, all in the same position: the left arm resting on the shoulder of the body before him. Whether this deposit testifies to sacrificial practices or a battle remains contentious[78,79]. Two horse specimens were sampled (GVA629_Fra_m112, and GVA636_Fra_m197) for DNA analysis and radiocarbon dating (UCIAMS250201, 2,105±20 uncal. BP, OxCal20 174-49 cal. BCE, and; UCIAMS250202, 2,125±20 uncal. BP, OxCal20 340-53 cal. BCE, respectively; Table S1).

*** France: Orcet, La Roche Blanche - L'Enfer, Department of Puy-de-Dôme**
[45.7076622, 3.17459]
The site of « l'Enfer », located on the communes of Orcet and La Roche Blanche, was discovered by François Baucheron and his team (Inrap) in 2002. Five pits were found, and yielded large numbers of horse complete skeletons, buried in groups of seven to 20 individuals. Both horses used in this study come from pit # F10. These horses are dated from the 1st century BCE, following radiocarbon dating (GVA637_Fra_m64: UCIAMS250203, 2,050±20 uncal. BP, 148 BCE-21 CE, and; GVA639_Fra_2: UCIAMS250204, 2025±15 uncal. BP, 51 BCE-55 CE; Table S1). The location of the site, near the gallic oppidum of Gergovia, where the

battle of 52 BCE took place. Therefore, the site may play host to some of the horses associated with this famous historical event[78,79].

*** France: Pech Maho**

[43.029047, 2.9808695]

The Pech Maho oppidum is located in the commune of Sigean (Aude, France), on the right bank of the River Berre, around ten kilometers west of the current shoreline of the Gulf of Lion, and around 20 kilometers south of Narbonne[80]. The site was occupied from the middle of the 6th century BCE, at a time when colonial trade was developing in the region, largely driven by the Greek cities of Massalia (Marseille) and Emporion (Ampurias). This small (1.5 hectares intra-muros), but heavily fortified coastal settlement, functioned for just over three centuries, both as a landing stage and as a particularly active trading centre, at the interface between the indigenous world and the Mediterranean world, maintaining special links with Roussillon, the Greek colony of Emporion and, more generally, with the Iberian world. A horse specimen (US78019, Cab-78-014) was sampled for DNA analysis and radiocarbon dating (Pech126_Fra_m288), and confirmed a date around the 4th and 3rd centuries BCE (UCIAMS250224, 2,225±20 uncal. BP, OxCal20 372-203 cal. BCE; Table S1).

*** Germany: Krefeld-Gellep**

E 51°19'55.8"N 6°41'03.4"E [51.3296206, 6.6612386]

The sampled horse skeleton from *Gelduba*, today Krefeld-Gellep in Germany, was found during the 2017/2018 excavation campaign in Nord *vicus* of the fort (Kref4_Ger_48; excavation identification number NI2017/0030 and archaeological context number 2708-12). It is one of nearly 200 horse skeletons discovered to date, demonstrating the result of several warlike conflicts[81,82]. The numerous horse and human graves without grave goods, diffusely scattered and intermingled over the site, indicate an unusual condition in burial rites. They prove on the one hand the "Revolt of the Batavi" in the autumn of 69 CE between the Romans and Batavi (Germanic tribe) and on the other hand incursion by the Franks in the middle of the 3rd century CE[81,83]. According to *Tacitus* (hist. 4, 26), Gelduba was the outermost outpost on the territory of the Ubians (Germanic tribe). It was located directly on the tributary of the Rhine, a natural part of the Limes in the province of Germania inferior, between the two important Roman cities of *Colonia Claudia Ara Agrippinensium* (today Cologne) and *Colonia Ulpia Traiana* (today Xanten). After the Revolt of the Batavi around 71 CE, the Romans started constructing the fort, including a vicus[81].

*** Germany: Rathewitz**

[51.14987, 11.80979]

Tomb No 13 was part of a burial ground located around Rathewitz (near Naumburg, Federal state of Saxony-Anhalt, Germany) and radiocarbon dated at the Migration period 5th-6th centuries CE[84]. A nearly complete skeleton of a stallion (Rat13_Ger_474; Inventar-Nr. LDA Halle: HK 56:281), and of a large male dog were found in this tomb. The age of the stallion was estimated of about 5 years. Its withers height was 134 cm which ranged in the middle of known body size variation for horses from this region and period[85]. Radiocarbon dating confirmed the context association with the Migration period (UCIAMS199237, 1,620±15 uncal. BP, i.e. 413-535 cal. CE; Table S1).

*** Hungary: Bakonszeg-Kádárdomb**

[47.17803, 21.46927]

The largest multi-layered '*tell*' settlement in the Berettyó River Valley in eastern Hungary on the Great Hungarian Plain[86]. Bakonszeg-Kádárdomb spans from the Early Bronze Age (EBA) Ottományi to the Middle Bronze age (MBA) Gyulavarsánd/Ottományi, dating from 1,748-1,624 cal BCE[87] (UCIAMS283904; Table S1). Horse BAK1_Hun_m1686, aged 11-12 y, was excavated from a refuse pit. The $^{87}Sr/^{86}Sr$ ratio indicates it was not local to this settlement, but likely originated from a site on the Danube near Budapest, such as Százhalombatta-Földvár, or Királyok Útja 293[20]. Comprised of mature animals between 1.28-1.39 meters at the withers (12.2-13.2 hh), the relative abundance of horses at Bakonszeg was high for the region (11.16%), signifying somewhat intensive use. Cattle were the principal large domesticate on site (43.6%), with relatively equal numbers of ovicaprids (22.5%) and pigs (22.75%).

*** Hungary: Berettyóújfalu-Szilhalom**
[47.2219014, 21.5135406845323]
Berettyóújfalu-Szilhalom is another *'tell'* settlement enclosed by a ditch, on a natural loess plateau in the Berettyó Valley, close to the town of Berettyóújfalu. Occupied from the Late Neolithic, the Bronze Age levels date to Early Ottományi and Classic Gyulavarsánd/Ottományi, between 2,150–1,450 BCE[88]. Horse BS11_Hun_m1682, aged 9-11y and dating to 1,740-1,623 cal. BCE (UCIAMS283905; Table S1), was found in a midden, and was not reared on the site, but in an area of higher radiogenic bedrock, probably from the nearby Füzesabony micro-region, or northeast into the foothills of the Bükk mountains[89]. Mostly mature animals were present, but juvenile horses suggest breeding on site, where horses comprised 3.66% of the faunal assemblage, which was dominated by pigs (37.0%), with equal numbers of cattle (29.7%) and ovicaprids (29.6%). Impressive cheekpieces for bridle bits were found in the uppermost occupation level, including one rod cheekpiece and one contamination form, in the same layer as a gold lockenring[90].

*** Hungary: Budapest-Királyok Útja 293**
[47.59933, 19.06793]
Probably a semi-fortified tell settlement, Budapest-Királyok Útja 293 lies along the right bank of the Danube in the Békásmegyer neighbourhood of Budapest, which was densely occupied in prehistory. The radiocarbon dates for five sample (collectively: 2,458-2,138 cal. BCE; Table S1) corroborate the known Early Bronze Age Bell Beaker-Csepel Group residency at the site. This gives way to Early Bronze Age Nagyrév (2,200/2,100–2,000/1,900 BCE) and Middle Bronze Age Vatya (1,800/1,700-1,600/1,500 BCE) levels, in line with the direct radiocarbon date on horse KU1701_Hun_m1822, genetically appearing as a DOM2 horse (1,895-1,749 cal. BCE). Horses KU1701_Hun_m1822 (6-7y), KU2210_Hun_m2218 (6-7y), KU709_Hun_m2335 (7-8y), KU1591_Hun_m2304 (7-8y), KU2102_Hun_m2211 (7-8y), and KU1153_Hun_m2301 (9-10y), all come from a large assemblage of horse bones recovered from buildings B and C, and from pits between them, with teeth and lower limbs overrepresented. Their genomic profiles and radiocarbon dates, however, indicate the succession of two horse lineages on the site, first local genetically, then DOM2-related, but they come from the same contexts. A lower leg and hoof were found articulated in a pit in the corner of a building, perhaps as a head-and-hoof offering[20]. The proximal phalanx in this deposit had a healed 'chip' fracture, a common malady of modern horses worked at high speeds. The care involved in healing this injury, and the evidence it recovered and continued to be used, suggests that riding, breeding use, or personal attachment, was seemingly greater than the use of this horse for carcass products. Bit wear was also recorded on two horses at the site, KU239/01 (6-7y), and KU C1.D-NY (9-10y). All of the horses but one were born locally, with KU2210_Hun_m2218 just outside the local mean, probably coming from the Füzesabony micro-region or the Great Hungarian Plain. Ranging from 1.23-1.30m tall (12-13.3 hh), young and very old animals were present at Királyok Útja 293, comprising a full breeding population. The age classes between 0-3 and 6-7 years were over-represented, potentially reflecting the culling young stallions prior to sexual maturity, alongside trade activities at the site.

*** Hungary: Dunakeszi-Székesdűlő**
[47.60788, 19.10966]
Excavations at Dunakeszi-Székesdűlő, which lies on a hill 500m east of the Danube, along the border between Budapest and Dunakeszi, yielded deposits from the Neolithic, Copper, and Bronze Age. Horses DS101_Hun_m2271, and DS113_Hun_m2222 come from refuse or storage pits from the Early Bronze Age Bell Beaker-early Nagyrév settlement layers, and were radiocarbon dated to 2,341-2,149 cal. BCE (Table S1). Ranging from 1.23-1.31m at the withers (12-12.3 hh), juvenile and adult horses were present, suggesting that breeding may have occurred on site, numbering 10.0% of the large domesticates, where cattle dominated the faunal assemblage (51.8%), along with ovicaprids (25.4%), and pigs (12.8%)[91]. The $^{87}Sr/^{86}Sr$ ratios for DS101_Hun_m2271, and another horse (DS396) not analysed genetically, corroborate that they were of local origins.

*** Hungary: Gáborján-Csapszékpart**
[47.22793, 21.65215]

Spanning the Early Bronze Age to the Middle Bronze Age Nyírség-Ottományi-Gyulavarsánd cultural periods, Gáborján-Csapszékpart is a '*tell*' settlement fortified with a ditch rising from the marshlands in the Berettyó River Valley on the Great Hungarian Plain by the village of Gáborján[86]. Cattle were the principal fauna (51.2%), followed by ovicaprids (22.9%), pigs (17.8%), and horses (3.9%)[92]. Three radiocarbon dates placed the occupation in the Early Bronze Age/Middle Bronze Age transition (2,291-1,830 cal. BCE, 2,200-1,954 cal. BCE, and; 2,131-1,784 cal. BCE)[87]. However, the two horses analysed genetically returned more recent radiocarbon dates spanning 1,870-1,622 cal. BCE (Table S1). Those two horses (GC77550_Hun_m1751, 12-18y, and; GC77486_Hun_m1681, 5-6y) were recovered from refuse middens, and were raised in the Berettyó Valley or in the nearby Füzesabony micro-region, according to isotopic signatures[20,89]. From the lower second premolar (LP2) of horse GC77.550, which exhibits a DOM2 genomic makeup, bitwear was identified[20].

*** Hungary: Százhalombatta-Földvár**

[47.339553, 18.946073]

Százhalombatta-Földvár is one of the largest, best known, and most extensively excavated tells, in a series of fortified Bronze Age settlements on the Danube[93]. Located on a high promontory on the western bank of the Danube about 30 kilometers south of Budapest, it has over 6 meters of habitation deposits, from Early Bronze Age Nagyrév (2,300/2,200 BCE), through the entire Middle Bronze Age Vatya period, 2,000/1,900-1,500/1,450 cal. BCE[94]. Horses SZHB2027_Hun_m2033, SZHB2074_Hun_m2115 and SZHB2147_Hun_m2054 (2,197-1,945 cal. BCE; Table S1) returned surprisingly early Nagyrév timelines, given that all the horses in this sample were thought to originate in the Middle Bronze Age Vatya deposits that were mostly recovered from the communal areas between houses[95], as confirmed through radiocarbon dating of the other additional horses also analysed genetically (except one, SZHB625_Hun_m984, 1,046-922 cal. BCE; Table S1).

The archaeology of the site has suggested full breeding population of horses, with neonates to very old (20+y) animals, fitting a transport profile, with the possibility of producing a few surplus animals for trade[20]. Withers heights from 1.32-1.496m (12.2-14.2hh), with the majority of horses born within the purview of the '*tell*', including SZHB553_Hun_m1566 (1-3y), SZHB2074_Hun_m2115 (3-4y), SZHB2147_Hun_m2054 (6-7y), SZHB625_Hun_m984 (6-7y), SZHB2027_Hun_m2033 (8-9y), SZHB734_Hun_m1576 (16-17y). The isotopic signatures of three horses, SZHB2158_Hun_m1820 (6-7y), SZHB967_Hun_m1822 (7-8y), and SZHB2079_Hun_m1575 (15-16y), suggested origins from elsewhere, probably in the Füzesabony micro-region[89]. There, the large Middle Bronze Age tell of Füzesabony–Öregdomb, had a high concentration of horses, twenty rod-shaped cheekpieces[96,97], and two strap distributors[98], while the Middle Bronze Age tell at Tiszafüred-Ásotthalom produced fourteen rod-shaped cheekpieces, one contamination form, five strap distributors, and probable riders in its cemetery at Tiszafüred-Majoroshalom. Thirteen rod-shaped cheekpieces have been excavated from Százhalombatta, from housefloors and general fill contexts across the site, including five Füzesabony types, and one triangle strap distributor. Bitwear was recorded on two horses, SZHB2361/3345 (10-11y) and SZH2313 (11-12y), which, however, were not analysed genetically[20]. Roughly 4 kilometers northeast, probable riders were also identified at the contemporaneous, related village of Érd-Hosszúföldek, a 20-25-year-old woman dating to 2,020–1,740 cal. BCE, and an 18–20-year-old male and a 35–40-year-old female, dating between 1,980–1,740 cal. BCE[20,99]. Strontium isotope analysis shows that the male was nonlocal and the females were local but spent their adolescence elsewhere[100], quite possibly in the Füzesabony micro-region, like the non-local horses, implying that horses and people may have been travelling together.

*** Iran: Dava Göz**

[38.4902778, 45.0322222222222]

Dava Göz is a tell settlement located north of the Lake Urmia, Iran, on fertile agricultural lowland, near the only passage from the Caucasus in the region. The original site extended over 2 ha but has been partly destructed due to subsequent agricultural activities. It now covers a surface of ~100 x 100 meters, and spans a time period from the Late Neolithic to the Chalcolithic phases of the local Dalma culture, i.e. approximately from 5,400 to 3,600 BCE[101]. The radiocarbon date obtained for the upper cheek tooth that was analysed genetically in this study (DavaG1_Ira_m4615, with archaeological indications Tr II, Loc 3003, RN3011) confirmed a Late

Neolithic timeline within the mid-5th millennium BCE (UCIAMS283903, 5,765±15 uncal. BP, i.e. 4,682-4,547 cal. BCE; Table S1). The site chronology and the exceptional quality of the excavation offer a unique opportunity to understand significant changes in the archaeological cultures and traditions in north western Iran, and their possible ramifications in the Caucasus, eastern Anatolia and Syro-Mesopotamia. Obsidian analysis indicated multiple sources until the Chalcolithic, a time when only one obsidian source was represented, possibly in relation to the subsistence economy shifting to transhumant livestock farming[102].

*** Israel: Sepphoris**

[32.745556, 35.278611]

The archaeological site of Sepphoris (Hebrew name *Zipori*, Arabic name *Saffuriya*), is located in the lower Galilee, ca. 6 kilometers northwest of the town of Nazareth. Occupation of the site spans many periods from the Hellenistic through to the Ottoman period[103,104]. Notable finds from the Roman period are a large theatre, monumental public buildings including a temple, numerous elaborate mosaics dating to the 3rd to 6th centuries CE, and remains of a water system including aqueducts which continued in use in the Byzantine period. In the Roman period, Sepphoris was the capital of the Galilee, and the most important Jewish center in the region. In the Byzantine period, it was a multi-ethnic city as attested by remains of a synagogue, dating to the first half of the 5th century CE, and two early Christian churches dating to the 5th or early 6th centuries CE. Later occupation of the site occurred in the Crusader and Islamic periods, with the site inhabited until 1948 when the Palestinian town of *Saffuriya* was depopulated. Sample MV243_Isr_294 returned two consistent radiocarbon dates overlapping the 3rd and 4th centuries CE (UCIAMS199270, 1,760±15 uncal. BP, OxCal20 242-346 cal. CE, and; UCIAMS199655, 1,750±20 uncal. BP, 242-375 cal. CE; Table S1).

*** Italy: Tarquinia**

[42.249167, 11.756111]

While the 'monumental complex' of Tarquinia opens a window into the cultural development of an Etruscan area sacred to the major female goddess of the Etruscans[105], the sample Tarquinia3206_Ita_275 analyzed in this study comes from superficial layers of the area surrounding the top curb of a hypogeum, which was obliterated in the 9th-10th centuries CE. It belongs to periods following the Etruscan, and confirms that the 'monumental complex' was in use for many centuries. Radiocarbon dating indicated that the horse died in the 3rd or 4th century CE (UCIAMS229407, 1,790±20 uncal. BP, OxCal 220-330 CE; Table S1).

*** Kazakhstan: Karatomar**

[52.804767, 62.839247]

The Karatomar burial ground, Kurgan 1, is located in the Kostanay region of northern Kazakhstan, on the left bank of the Tobol branch of the Karatomar reservoir. The kurgan was erected in the Bronze Age and belongs to the Sintashta Culture. Burial Pit 4 was robbed in antiquity. There was evidence of overlapping of birch and pine logs covering the burial, with horse mandibles fixed on the overlap. The walls of the burial were lined with planks. Two small vessels were found in the burial and under the floor of the chamber (at a depth of 3 meters) a "cache" was discovered, which included a bronze vessel containing bronze axes[106]. Radiocarbon dating confirmed that the horse specimen analysed genetically (Karat17039_Kaz_m1834) lived during the early second millennium BCE (UCIAMS283909, 3,515±20 uncal. BP, i.e. 1,918-1,750 cal. BCE; Table S1).

*** Moldova (or Moldavia): Odaia**

[47.987105, 27.788329]

The archaeological site of Odaia is located close to the village Miciurin, County Drochia, Republic of Moldova (or Moldavia). It was a Late Bronze Age settlement of the Noua-Sabatinovka-Coslogeni culture. This culture occurred between the Dnepr River in the East and the eastern part of Transylvania in the West and has been dated from 1400 to 1100 cal. BCE[107]. A fraction of approximately 99% of about 20,000 bones found at this site were from domestic animals. Although cattle were the dominant animals, a total of 792 bones or bone fragments (representing ~4%) were from horses. The sample collected for DNA analysis (Miciurin01_Mol_794) was, however, intrusive as radiocarbon dating returned a calibrated age of 707-880 cal. CE (UCIAMS199239, 1,225±15 uncal. BP; Table S1).

*** Mongolia: Biluut-2 (Khuiten Gol Delta), Pazyryk Burials 2-1 and 2-2**

[48.631233, 88.358433]

This site, located along the northern shore of Khoton Lake in far western Mongolia, consists of a large looted cemetery linked to the Pazyryk culture (ca. 400-200 BCE). Two looted mounds excavated during a joint endeavor of the Smithsonian Institution, the National Museum of Mongolia, and Eastern Tennessee State University between 2011-2013 produced horse remains alongside wooden coffins, heavily degraded horse equipment (jointed iron snaffle bits, argali-shaped gold cheekpiece covers), and ceramics displaying organic residue. Feature 2-1, containing the burial of an older male horse roughly 14-15 years of age (estimate based on crown height; M17x152x1_Mon_m80), showed osteological evidence of use in transport including bit wear and premaxillary changes linked with heavy exertion. Another burial, Feature 2-2, contained the skeleton of a juvenile horse, likely between 2-2.5 years of age at the time of death based on tooth eruption (sample M17x156_Mon_m299; UCIAMS190017, 2,250±15 uncal. BP, 389-208 BCE; Table S1).

*** Mongolia: Egyin Gol**

49° 27 N, 103°30E [49.45, 103.5]

The necropolis of Egyin Gol is located north of Mongolia at the confluence of Eygin Gol and Selenga River, in an environment of forests and grasslands of the peri-Baikal area where herds of cattle and horses are plentiful. It is, thus far, the only Xiongnu burial site fully excavated[108]**.** It comprises a total of 108 tombs, dated between the 3rd century BCE and the 2nd century CE[109]. The site is divided into four sectors according to different cultural and economic influences. The offerings of horses and cattle found within the tombs reflect the social position of the human individuals buried. A total of four horse remains were sampled for DNA analyses and radiocarbon dating (samples EGI10_Mon_17, EGI12x2_Mon_14, EGI67_Mon_14 and EGI69_Mon_75; Table S1). Radiocarbon dates (UCIAMS250209-UCIAMS250212) spanned the time period between 46 cal. BCE and 124 CE (Table S1).

*** Mongolia: Tamir, tomb nb. 13 and 22**

[47.76691, 102.45019]

The necropolis of Tamir (Tamiryn Ulaan Khoshuu) is located west of the confluence of the Tamir and Orkhon Rivers in central Mongolia. It spans nearly 22 hectares at the foot of a large granite outcrop overlooking the floodplain of the Tamir River in a steppe-like environment. The necropolis is divided into two main areas, a western sector (344 tombs) and an eastern sector (54 tombs), both separated by a ravine. The eastern sector is the only sector to have been excavated. It comprises large (elite) tombs dated from the 1st century BCE to the end of the 1st century CE. Genetic analysis revealed the presence of a prominent genealogy with two familial lines, A and B, with ten and nine individuals respectively distributed over five to six generations[110]. The site differs significantly from Eygin Gol as it displays more Chinese affinities. Offerings of goats are frequent, those of horses rarer. The remains from two horses were selected for DNA analyses and radiocarbon dating (TAM13_Mon_14 and TAM22xA2_Mon_5). Radiocarbon dating confirmed that the two horses located in tomb nb. 13 and tomb nb. 22 both lived at the transition between the 1st century BCE and the 1st century CE (UCIAMS250215, 2,000±20 uncal. BP, OxCal20 46 BCE-73 CE, and; UCIAMS250216, 2,015±15 uncal. BP, OxCal20 48 BCE-58 CE, respectively; Table S1).

*** Mongolia: Burgast, Bayan Ölgii province**

[49.830833, 89.99]

The Burgast site is located in the Mongolian Altai. It was excavated in 2015 and 2016 by the French archaeological mission in Mongolia (dir. Sébastien Lepetz). Among archaeological structures from different periods (ranging from the 10th century BCE to the 10th century CE), several tombs and ritual structures from the Türk period were discovered. Among them, a pit (ST 8), dated to the 7th-8th centuries CE, yielded the remains of a single horse, from which a sample was taken for DNA analysis (GVA9046_Mon_716)[111].

*** Mongolia: Zoolongiin am 1-1, catacomb №2**

48°23'20.51"N 101°21'14.46"E [48.3890306, 101.354016666666]

The archaeological complex Zoolongiin am 1-1 is located in the Erdenemandal District (somone) of the Arkhangai Aimag (Mongolia), 15 kilometers south-east of the district center. The excavations were carried out in 2017 by a Russian-Mongolian expedition co-led by A. A. Kovalev, T.-O. Iderkhangai and D. Erdenebaatar[112]. Three catacombs with vertical entrance shafts and chambers were let into the mound of the Bronze Age mound, at a depth of about 1–1.5 meters. The entrance shafts of the catacombs were densely packed with earth and stones. Two horse skulls were found in catacomb No. 2, and were investigated in this study. One skull was placed in the upper part of the shaft (Zoox1_Mon_333), and the second skull (Zoox2_Mon_271) was in the chamber of the catacomb), along with the remains of some wooden object. Both specimens were radiocarbon dated to 215-407 cal. CE (UCIAMS208901, 1,710±15 uncal. BP, 258-407 cal. CE, and; UCIAMS210915, OxCal20 1,800±20 uncal. BP, OxCal20 215-326 CE, respectively).

*** Poland: Tominy**
[50.853036, 21.679608]
Two horse teeth were sampled from a food waste bone assemblage including 8 specimens, which as recorded in the feature No. 115 of an Early Neolithic settlement (Linear Pottery culture; 6th/5th millennia BCE). This feature was disturbed by a younger pit grave of a Funnel Beaker culture. Cattle and pig remains also belonged to the assemblage. Homogeneous faunal material of the Linear Pottery culture consisted mainly of cattle, red deer and the horse. The latter was absent in the Faunal Beaker culture assemblage (the 4th and 3rd centuries BCE). AMS radiocarbon data for object 108 with animal bones, including 10 horse bones, indicated at the time of deposit creation around 5,217–5,000 cal. BCE (95.4%) (Poz-49591 6,160±40 uncal. BP)[113]. Radiocarbon dating of the two horse remains analysed in this study (POZ327_Pol_m5127 and POZ37_Pol_m5108) returned similar timelines (UCIAMS283914 and UCIAMS283915, 6,165±15 and 6,150±15 uncal. BP, i.e. 5,210-5,066 cal. BCE; Table S1).

*** Russia: Krasny Gorodok settlement, Abashevo**
[50°51'10.93" N, 21°40'46.59" E; 53.6536111, 50.6322222222222]
The Krasny Gorodok settlement site is located in the Samara region, on the left bank of the Sok river, the right tributary of the Volga river. The site contained a stratigraphic layer associated with Abasheva cultural finds, dating back to the 22nd-19th centuries BCE[114]. However, despite originating from this excavation layer, the horse remain sampled for this study (ABA3_Rus_1333) returned radiocarbon dates overlapping the 13th and 14th centuries CE (UCIAMS250364, 675±15 uncal. BP, OxCal20 1,281-1,385 cal. CE; Table S1). The remain was, thus, intrusive and was in fact contemporary to the Golden Horde period, founded by Turks, following the westward expansion of Genghis Khan's descendants.

*** Russia: Arzhan-2, burial nb. 5**
[52.096003, 93.711080]
During the excavations of 2001, more than 5,700 gold items were discovered in the undisturbed royal burial nb. 5. Arzhan-2 is located in a plain where hundreds of kurgans are arranged in alignments. The kurgan Arzhan 2 is one of the largest monuments of that kind excavated ever; it is nearly 80 meters in diameter and more than 2 meters high, with a platform constructed from stone slabs, which is characteristic of the early Scythian period[115]. Excavation yielded numerous human remains, different archaeological materials including organics, and horse remains largely associated with grave 16. Based on dendrochronology and radiocarbon dating, its age is commonly accepted as belonging to the 7th– 2nd centuries BCE[116]. The materials of Arzhan-2 show multiple cultural parallels with the neighboring territory, which reflects broad cultural relations with different areas beyond Tuva region, that is in Kazakhstan, Mongolia, and Northern China[117,118]. Sample Rus8_Rus_855 was radiocarbon dated to 900-809 cal. BCE (OxCal20, UCIAMS182711, 2,700±20 uncal. BP).

*** Russia: At Daban 13**
61°39N, 129°15E [61.651208, 129.253549]
The site of At Daban is located on a Quaternary terrace overlooking the Lena River near Yakuts (North Siberia, Sakha republic). It is the burial place of the Kangalaski clan, who ruled Yakutia from the late 17th century CE

to the early 19th century CE, and formed a middle ground alliance with the Russians[119]. Horse offerings are standard in Yakut tombs, and the tomb that provided the horse material analyzed in this study dates from an early chronological phase of the burial complex, corresponding to the first half of the 18th century CE[120] (ATDABAN13_Yak_1725).

*** Russia: Brusyany IV**
[53.208019, 49.395187]
The site of Brusyany IV corresponds to an Early Middle Age site. Two horse specimens (LR18x68_Rus_592 and LR18x70_Rus_608) were sampled for DNA analysis and radiocarbon dating (UCIAMS250250, 1,495±20 uncal. BP, OxCal20 547-636 cal. CE, and; UCIAMS250251, 1,465±20 uncal. BP, OxCal20 571-644 cal. CE, respectively; Table S1).

*** Russia: Chekon**
[45.1161778, 37.49716111111111]
The Chekon settlement is located in the Krasnodar Territory, near the city of Anapa, Russia. The settlement stands on the left bank of the river. The area of the excavation was 6,300 square meters, and revealed a settlement dating back to the 4th millennium BCE, affiliated to the Maikop culture. However, the horse sampled for this study (KUZ3_Rus_m954) was found to be intrusive as it delivered a more recent dates overlapping the whole 10th century BCE (UCIAMS250205, 2,795±15 uncal. BP, OxCal20 1003-904 cal. BCE; Table S1).

*** Russia: Derkul**
[51.16, 51.17]
Derkul is a settlement dating back to the Neolithic, located in the Orenburg region of Russia. The excavation at Derkul has revealed a single house-like structure. The design of the structure and the construction methods provide clues about the lifestyle, social organization, and cultural practices of Neolithic population from this region. Both Neolithic artifacts and animal bones, including the horse remain sampled in this study (NB4_Kaz_m4210), could be unearthed from this small site (UCIAMS250233, 5,270±20 uncal. BP, OxCal20 4,429-3,991 cal. BCE; Table S1).

*** Russia: Filippovka II, kurgan 1, grave nb. 2**
N51°20'45,12" E54°04'33,38" [51.345867, 54.075939]
The bones of the horse that was sampled for this study (LR18x84_Rus_m294) were found as part of the filling of a burial that belonged to Kurgan cemetery, which is affiliated to the Sarmatian culture, and dates back to the 5th-3rd centuries BCE[121]. The horse was radiocarbon dated to the 4th-3rd centuries BCE (UCIAMS218285, 2,230±30 uncal. BP, OxCal20 387-200 cal. BCE; Table S1).

*** Russia: Idzhil-I, kurgan 1, Kalmykia**
[47.42, 45.523]
Sample IDZH_Rus_734 of a horse bone comes from an Early Medieval grave that was located under burial mound (kurgan) 1 in the kurgan burial ground Idzhil-I situated in the interfluve of the Lower Volga River and its former seasonal distributary Sarpa River in the northern territory of the modern Republic of Kalmykia (Russian Federation). This site is located in an arid desert-steppe landscape, which was historically populated by seasonally migrating pastoral nomads, who buried their dead in dispersed graves. Idzhil-I kurgan group was rescue-excavated in 1981 and had only one grave of the early Khazar Period (7th-8th centuries CE). This grave contained an elite male nomad, who was buried with the head and legs of the ritually-tacked male horse. The horse sample was radiocarbon dated to 670-798 cal. CE (OxCal20, UCIAMS218233, 1,270±20 uncal. BP; Table S1), in line with the archaeological context.

*** Russia: Industryia**
[43.862852, 42. 704325]
Industriya 1 is an Early Iron Age cemetery at the outskirts of the mineral spa Kislovodsk, Russia. The site is located at the slope of a gorge and covers an area of at least 150 to 150 meters. Burials in stone boxes are

regularly washed out by erosion. The horse burial was recorded in 2001 by Dmitry S. Korobov[122]. It was partially destroyed horse skeleton without traces of a burial pit. The horse was harnessed with a full gear including bits, psaliae and several bronze and bone buckles of pre-scythian typs. Bone and bronze arrowheads as well as remains of a scale armor were found near the horse. Typologically, the horse gear could be earlier than the date in late 8th century BCE, yet dating's from the nearby settlement and most of the burials support such a dating[123]. Direct radiocarbon dating indicated that the horse analysed in this study (KAU27B_Rus_m627) lived between 765 and 489 cal. CE (UCIAMS250214, 2,475±20 uncal. BP; Table S1).

*** Russia: Ipatovo 3, Kurgan 2, Animal Complex 13**

[45.676606, 42.915092]

The big mound of Ipatovo (Ipatovo 3, mound 2) was part of a series of linear alignments of burial mounds that run west-east crossing the of the Kalaus river, Russia. Today the slightly hilly zone around the site is part of the herb and grass-steppe zone, while the Kalaus valley itself is part of the desert-steppe. The site was excavated 1998 to 1999 during rescue excavations by the heritage organization "Nasledie" (direction: A.B. Belinskiy, A.A. Kalmykov). Mound 2 was the largest in a group of at least eleven mounds visible from aerial images and yielded a total number of 195 burials in 11 construction phases. Of the interments, a total of 151 are associated with a 18th century CE cemetery of nomadic Nogay people. One grave held the burial of a splendidly furnished Sarmatian woman, two were Iron Age, and 34 graves are assigned to a Bronze Age date[124]. The first 4 building phases are associated with burials of the 4th mill. BCE. Construction phases 5-11 have been associated with early and late catacomb grave interments dating to the 3rd millennium BCE, some of which with wooden wagons. Into this mound four post-catacomb and 9 Late Bronze Age graves were added. To the later belong "animal complex 13", which is part of this study. Graves 5 and 8 overlap in dating with this complex but are not located nearby. The animal bones were found in the northwestern sector in the fill of ditch 3, but without indications for a pit. They comprised a horse scull scattered in pieces in an area of 1,5m, which provided the material for this study (KAU22_Rus_m877). The age of the horse is estimated to be 16-17 years. Radiocarbon dating indicated that the horse lived between 922 and 832 cal. BCE (UCIAMS250213, 2,745±15 uncal. BP; Table S1).

*** Russia: Katanda-II, The "Big Katandinsky" Kurgan**

50°10'05.2"N 86°10'12.4"E [50.1681111, 86.170111]

The so-called "Big Katandinsky" mound is located in Altai, in the vicinity of the village of Katanda (Ust`-Koksinsky District of the Altai Republic, Russia). It was partially excavated in 1865, under the leadership of V. V. Radlov, as part of the larger archaeological complex Katanda-II. The preliminary excavation was reopened by A. A. Gavrilova in 1954, in the framework of the Katandinsky detachment of the Gorno-Altai expedition of the State Hermitage[125]. The horse burials between the north wall of the burial chamber and the north wall of the grave pit represented important discoveries[126]. They comprised the remains of 22 horses, deposited in three layers with nine skeletons in the lower layer, eight skeletons in the middle layer, and five skeletons in the upper layer. The exact position of each animal was not recorded at the time of the excavation. Skeletal marks indicate that horses were killed by a blow to the skull. Few items of horse equipment were identified. The mound dates back to the Scythian-Saka time of the 4th–3rd centuries BCE, and belongs to the Pazyryk culture, which was somehow confirmed by radiocarbon dating of two remains (Kat2x4_Rus_m112, horse 15: UCIAMS250221, 2,105±20 uncal. BP, OxCal20 174-49 cal. BCE; Katx11_Rus_m269, horse 7: UCIAMS250222, 2,200±15 uncal. BP, OxCal20 360-178 cal. BCE; Table S1).

*** Russia: Khankarinsky Dol (Mound group), Mound No. 11**

51°19'3.59" N; 83° 2'40.79" E [51.3176639, 83.0446638888888]

The mound group of Khankarinsky Dol belongs to the Pazyryk culture. It is located on the second terrace (above the floodplain) of the left bank of the Inya river, a left tributary of Charysh, approximately ~1.2 kilometers southeast of the Chineta village (Russia, Altai Krai, Krasnoshchyokovsky District, Northwestern Altai). The site has been investigated since 2001 by the Altai State University archaeological expedition led by P. K. Dashkovsky[127]. Mound No. 11 is found in the central part of the burial ground. The diameter of the

mound, made of 1–2 layers of stones, was 7.25 meters. The height of the construction reached 40 centimeters above ground level. Along the perimeter of the barrow, there was a circular mound, with a grave at the center (dimensions = 2.85 meter × 2.2 meter × 1.96 meter). It contained the remains of a wooden structure, and the bone remains of a 20-25 years old woman. She was found lying on her right side at the southern wall, bent over, with her head oriented to the east. A burial of a horse was also found along the northern wall of the grave pit. The animal (sample Han12_Rus_m296) was positioned on its stomach, with legs tucked in, and its head facing toward the buried woman. This burial contained silver earrings, a copper mane, covered with gold foil, a bronze mirror, iron bits, a ceramic jug, scraps of gold foil and "meat food" (lamb). Kurgan No. 11 dates back to the 4th–3rd centuries BCE, as confirmed by radiocarbon dating (UCIAMS250219, 2,240±15 uncal. BP, OxCal20 383-208 cal. BCE; Table S1).

*** Russia: Krasnosamarkskoe IV, kurgan and settlement**
[53.0871011, 50.809757] and [53.0936111, 50.8036111]
Two horse remains were sampled from the bone assemblages found at the Krasnosamarkskoe IV kurgan and settlement sites (RN10_Rus_1131 and RN85_Rus_865, respectively). Despite being associated to a Srubnaya cultural level (1,900-1,700 BCE), radiocarbon dating returned considerable younger age estimates spanning the late 8th to the early 13th centuries CE (UCIAMS229405, 905±20 uncal. BP, OxCal20 1,045-1216 cal. CE, and; (UCIAMS208886, 1,160±15 uncal. BP, OxCal20 772-957 cal. CE, respectively; Table S1). The two horses investigated at the DNA level corresponded, thus, to intrusive material from the Middle Ages.

*** Russia: Sadgorod IV, kurgan 2, sacrificial complex 2**
N 53°22'44,4" E 51°11'41,0" [53.379, 51.194722]
Kurgan nb. 2 from Sadgorod IV delivered a series of graves affiliated to the Srubnaya culture[128]. The horse specimen that was investigated in this study (LR18x5_Rus_1810) comes from the remains of a complete skeleton located in separate pit. Radiocarbon dating indicated that the remains in fact belonged to an animal that died by the late 17th century CE or later; it, thus, had no relationship with the Srubnaya culture (UCIAMS218274, 105±30 uncal. BP, OxCal20 1,683-1,936 cal. CE; Table S1).

*** Russia: Shumaevo I, kurgan5**
N51°45'50,3" E52°54'16,7" [51.763972, 52.904639]
Kurgan 5 at Shumaevo I dates back to the Golden Horde, from the 13th century CE to the first quarter of the 14th centuries CE. Radiocarbon dating of the two specimens analyzed in this study (LR18x65_Rus_1352 and LR18x66_Rus_1353) confirmed this cultural affiliation (UCIAMS218283, 610±30 uncal. BP, OxCal20 1,299-1,404 cal. CE, and; UCIAMS218284, 605±30 uncal. BP, OxCal20 1,300-1,406 cal. CE, respectively; Table S1).

*** Russia: Tyubyak**
[52.826215, 56.059210]
The settlement of Tyubyak, Russia, included two Bronze Age stratigraphic layers. The earliest layer corresponds to the Abashevo culture, and was covered by the more recent Srubnaya layer. The radiocarbon dates returned for samples ABA9_Rus_m1786 and ABA10_Rus_m1757 indicate that both samples belong to Late Bronze Age Srubnaya culture (Table S1; UCIAMS263094 and UCIAMS263095, 3,460±15 and 3,430±15 uncal. BP, respectively, i.e. 1,878-1,694 and 1,871-1,642 cal. BCE), which fits the overall 1,750-1,550 BCE timeline determined on the territory of the Volga-Ural region[129].

*** Russia: Yaloman-II, mound No. 46a**
50°31'19.5"N 86°34'00.2"E [50.5220833, 86.5667222222222]
The ancient necropolis of Yaloman-II is located at the mouth of the Bolshoy Yaloman River (the left tributary of the Katun River) in the Ongudaysky District of the Altai Republic (Russia). It dates to the Xiongnu time (2nd century BCE – 1st century CE), and belongs to the Bulan-Koba archaeological culture of the Early Iron Age. Excavations were carried out by an expedition of Altai State University under the leadership of A. A. Tishkin in 2003[130]. Mound No. 46a was discovered during a continuous opening of the site. A large horse skull and the

rest of the skeleton bones, as well as items of horse equipment, were found in the grave pit. The main occupation of the population of the Bulan-Koba culture was nomadic (yaylazhno-gornodolinnoe) animal husbandry. The sample collected for DNA analysis (Yal2x24_Rus_m105, #M34) was radiocarbon dated to 166-44 cal. BCE (OxCal20, UCIAMS250255, 2,085±20 uncal. BP; Table S1).

*** Russia: Zayukovo-3 burial ground, sacrificial pits 7 and 8**

[43.61611, 43.33389]

The Zayukovo-3 burial ground is located near the village of Zayukovo in the Baksan district of the Kabardino-Balkar Republic. To date, a total of 301 burials have been found on the monument. The burials date from the 8th century BCE to the 7th century CE. Sample OSCAE16xSP7x1_Rus_604 was excavated from the sacrificial pit nb. 7, and belonged to a horse carcass that was littered with ragged fragments of limestone. Sample OSCAE16xSP8_Rus_577 was excavated from the sacrificial pit nb. 8 of the Zayukovo-3 burial ground. The sacrificial pit was oval-shaped, and, contained a bull lay together with the bones of a horse. Both animals were represented by their skulls and leg bones only, but skin may have also been deposited. Radiocarbon dating indicated that both horses died in the mid 6th – mid-7th centuries CE (UCIAMS190021, 1,475±20 uncal. BP, OxCal20 565-642 cal. CE, and; UCIAMS190022, 1,500±15 uncal. BP, 550-603 cal. CE, respectively; Table S1).

*** Slovenia: Bled, Pristava, grave nb. 18**

[46.357236, 14.093322]

The horse remain SRSLO012_Slo_m197 was excavated in the Pristava necropolis close to Bled (Slovenia), and was associated to grave nb.18. A large number of animal bones were found close to the graves, mainly above the cover, but not inside (Gabrovec 1960, 23-24), and this was also the case for the molar considered in this study. In general, the assemblages from the Pristava necropolis are dated by St. Gabrovec to "*the early Ha C period with considerable elements preserved from the Ha B culture*". A bronze spiral-headed needle and a ceramic Stillfried-Hostomice cup were found inside grave nb. 18. Both are indicative for the end of the Late Bronze Age (Ha B3), but can be also found at the very beginning of the Iron Age period (Ha C). Therefore, the grave was provisionally dated between the 9th and the 8th centuries BCE (pers. com. Peter Turk). However, radiocarbon dating of the molar points to the late Iron Age, which indicates that it was intrusive in the described context (UCIAMS250232, 2,125±20 uncal. BP, 340-53 cal. BCE; Table S1).

*** Spain: Alorda Park, Tarragona**

[41.190592, 1.580915]

Sample H9020_Spa_m291 was retrieved from the deepest levels of filling of the ditch that protected the fortified settlement of Alorda Park, Calafell, Tarragona). It was an isolated tooth (4th premolar) that was mixed with abundant faunal remains corresponding to the main domesticated mammals (*ie.* cattle, pigs and caprines[131]). The level corresponds to the 4th century BCE, in line with the radiocarbon dating of the specimen to 376-205 cal. BCE (OxCal20, UCIAMS190014, 2,230±15 uncal. BP; Table S1).

*** Spain: Can Roqueta-Torre Romeu, Sabadell, Barcelona**

[41.422930, 2.128527]

Sample CRTR279_Spa_506 was extracted from a skull preserving both maxillae located in a Late Roman silo (5th-6th centuries CE) at the site of Can Roqueta-Torre Romeu, in the northeast of the Iberian Peninsula (Sabadell, Barcelona, Spain). The late Roman sector of Torre Romeu, in the archaeological site of Can Roqueta, is interpreted as a small rural settlement. A total of 75 structures, silos and structures for habitation and wine and pottery production were excavated in one hectare. The general chronology of the Torre Romeu sector ranges from the ancient Neolithic to the late Roman period.

*** Spain: El Graell, Vic, Osona, structure E-42**

[41.922642, 2.242059]

Sample ADNUB33_Spa_m64 was extracted from a complete hemi-mandible located in a silo of the Iberian Culture (Roman phase, 1st century BCE-1st century CE; Table S1) at the site of El Graell, in the northeast of the Iberian Peninsula (Vic, Osona, Spain). The site was interpreted as a small rural settlement. The general

chronology of El Graell ranges from the Early Bronze Age to the Late Middle Ages. A total of 126 structures were excavated over 20.11 hectares, most of which correspond to silos.

*** Spain: Hereuet, Lleida**
[41.881866, 1.110246]
The rescue excavation performed at Hereuet (Serós, Lleida) revealed the remains of a small rural settlement that comprised several rooms and underground silos for cereal storage[132]. The equid analysed here (sample H2012x137_Spa_m204) was deposited in Silo nb. 8, in partial anatomical connection, together with other animals (two other equids, a cattle and a dog) during the Late Iron Age. Radiocarbon dating indicated that the animal died 350-58 cal. BCE (OxCal20, UCIAMS250253, 2,150±20 uncal. BP; Table S1).

*** Spain: Puig de Sant Andreu, Girona**
[42.005664, 3.07836]
With a known area of approximately 10 hectares, Puig de Sant Andreu is the biggest urban indigenous site in the Empordà area (Ullastret, Girona). Sample UE14029_Spa_m293 was found in the stratigraphic unit SU14029, which corresponds to an abandonment layer dated from the beginning of the 2nd century BCE[133]. Radiocarbon dating confirmed the archaeological contexts, returning a calibrated date of 380-206 cal. BCE (UCIAMS190016, 2,235±15 uncal. BP; Table S1). The level preserved several disarticulated faunal remains corresponding to the main domesticate mammals, mostly caprines.

*** Sweden: Eketorp, Gräsgård parish, Öland**
[56.295418, 16.48703]
The site corresponds to a waterhole sacrificial site, containing animal bones[134]. It is located just outside Eketorp ringfort, which was in use during the first 11 centuries CE (*ie.* 0-1100 CE). Sample F612_Swe_495 was radiocarbon dated to 425-565 cal. CE (OxCal20, UCIAMS218302, 1,570±30 uncal. BP; Table S1).

*** Sweden: Skedemosse, Gärdslösa parish, Öland**
[56.831197, 16.754477]
The site corresponds to a wetland sacrifice site containing weapons as well as animal and human bones. It functioned as a ritual place from the Pre-Roman Iron Age into the Late Viking Age, approximately 500 BCE-1050[135]. Sample F139_Swe_342, which was analysed genetically, was radiocarbon dated to 255-428 cal. CE (OxCal20, UCIAMS218299, 1,685±30 uncal. BP; Table S1).

*** Tajikistan: Shohidon, grave №20**
38°37'09.7" N; 69°58'45.7" E [38.6193611, 69.97936111111112]
The Shohidon burial ground was discovered by local residents in 2011, on the outskirts of Sari-Khosor village, in the valley of the Surkhob mountain river (Baljuan District, Khatlon province, Republic of Tajikistan). The graves were located on a loess outlier, whose soil was used to make building blocks[136]. In the period from 2012 to 2015, the Tajik-Russian expedition, led by T. G. Filimonova, carried out inspections and rescue excavations[137]. More than 30 burials were discovered and recorded, many of which already destroyed or looted. In 2019, A. A. Tishkin conducted an additional inspection of the damaged monument and of the finds stored in the local village museum[138]. Grave № 20 consisted of two chambers, separated by a stone lining that covered the entrance of the second chamber. The first chamber contained the burial of a horse, whose skeleton was completely preserved and found in anatomical connection. The animal was laid on its belly with legs tucked up, and iron bits were found in its mouth. Behind the stone lining, there was a burial of a male warrior lying on his back, with his legs tucked up. He had the following funerary equipment: a knife, a sword, arrowheads and two stirrups (all made of iron), as well as a one-handed pottery jug. The horse remain that was tested for DNA (Shohx1_Rus_720) was radiocarbon dated to the end of 7th century and the first half of 8th century CE (UCIAMS250235, 1,295±20 uncal. BP, 664-775 CE). Excavations of early medieval necropolis Shohidon allowed researchers to understand important features of interethnic integration of nomadic and sedentary agricultural populations in the Surkhob river valley.

*** Türkiye: Çadir Hoyük**

[39.676776, 35.1436]

Specimen CD5041_Tur_m314 derives from Çadır Höyük, a small mound site located in Yozgat province on the central Anatolian plateau with deposits dating from the Chalcolithic (c. 5,000 BCE) through the Byzantine and Seljuk periods. The specimen was recovered in 2013 from trench NTN7, locus 51 (FCN12830) from the North Terrace, an area located adjacent the main mound and the site of an agricultural estate in the and Roman and Byzantine periods. The find context represents a sealed courtyard surface with paving stones beneath a late Roman period structure. The equid specimen, identified as a first or second mandibular molar, exhibits classic caballine dental morphologies. A direct radiocarbon assay places it in the late 4th century BCE (c. 310 BCE) (Hellenistic period; UCIAMS250208, 2,275±15 uncal. BP, OxCal20 396-232 cal. BCE; Table S1), and represents the earliest dated material from the Terrace area, pushing the occupation of the Terrance significantly earlier than the late Roman and Byzantine architecture under which it was discovered.

*** Türkiye: Yenikapı, Istanbul**

[41.00521, 28.95172]

The specimen labeled KSK11b_Tur_829 represents a disk of bone cut in the medial-lateral direction from the metapodial shaft of an equid (including a prominent nutrient foramen). This specimen derives from Yenikapı Metro excavations, Istanbul dating to the Byzantine period and was originally sampled and exported for aDNA analysis in 2015. Specimen KSK11b_Tur_829 has produced a ^{14}C assay of 1,210±20 uncal. BP (UCIAMS199615), congruent with the Byzantine date of the Yenikapı equids. For the record, this specimen was mislabeled while in the process of being sampled for isotopic analyses in 2016 (KSK normally stands for Köşk Höyük).

*** Ukraine, Husatyn**

[49°04'31.2" N, 26°10'45.1" E; 49.075333, 26.179194]

The petrous bone labeled POZ54_Ukr_m1498 was taken from a skeleton of double-buried horses in a round barrow. It was discovered in a field near Husiatyn, Ternopil Oblast, in western Ukraine on a high plateau surrounded by the steep right bank of the Zbruch River, a left tribute of the Dniester. The burial consisted of two males. The first horse, located on the western side, was 13-15 old, and in height at the wither of 134–138 centimeters (mean = 136 cm). The second horse 2, located on the eastern side, which was genetically analysed, was 15-16 old, and showed a size at the wither of 148.2–154.6 centimeters (mean = 151.7 cm). Radiocarbon dating indicated that the time of deposition was 1,540–1,455 cal. BCE (UCIAMS283916; Table S1), in line with the two radiocarbon dates previously obtained (Poz-89334, 3,230±30 uncal. BP, and; Poz-107626, 3,185±30 uncal. BP)[139]. The horses' bodies were placed on their sides in a mirror-image position. The autopodial segments of the forelimbs were drawn up, while the hind limbs were extended. The examination of their bones and teeth shows that the roles and positions of these horses were not equivalent. The traces left on the incisors of Horse 2 suggested that it had been particularly important and enjoyed its masters' special favour.

Section 2. Radiocarbon Dating

The vast majority of the specimens analyzed in the present and previous studies was radiocarbon dated at the Keck AMS laboratory, University of California Irvine (USA), with the following methodology. Samples of cortical bone were cleaned mechanically and aliquots of ~200mg were crushed to mm-sized chips. If contaminating conservation materials were present, samples were sonicated in acetone, methanol and ultrapure MQ water in a water bath cooled to well below the melting point of collagen. Bone was decalcified overnight at room temperature, using a measured amount of 1N HCl just sufficient to dissolve all of the bone mineral, if no collagen was present. The demineralized samples were washed with MQ water and gelatinized overnight at 60°C and pH 2, ultrafiltered in precleaned Vivaspin 15 devices to select the >30kDa molecular weight fraction, and freeze dried overnight. Aliquots of 2mg of collagen were combusted under vacuum in quartz at 900°C with CuO and silver wire and the resulting CO_2 was cryogenically purified and graphitized on Fe by hydrogen reduction for ^{14}C measurement by AMS on an NEC 0.5MV. 0.7mg collagen aliquots were sealed in tin capsules and flash combusted in a Fisons NA1500NC elemental analyzer interfaced to a Finnigan Delta Plus isotope ratio mass spectrometer for elemental analyses and $d^{13}C$ and $d^{15}N$ measurements.

Additionally, horse remains from Ulaan Tolgoi and Zunii Gol[140] and Biluut[141,142] were dated using accelerator mass spectrometry (AMS) at Beta Analytic following laboratory protocols (https://www.radiocarbon.com/carbon-dating-pretreatment.htm), while horse remains from Zeerdegchingiin Khoshuu and Zuunkhangai were dated at the AMS Laboratory at the University of Arizona, following protocols outlined by Taylor et al (2017)[143]. Briefly, samples were surface cleaned and rinsed for one hour in an ultrasonic water bath. Exterior and cancellous portions of the sample were removed with a sterilized drill bit, before crushing and sieving to produce a 500 mg sample with uniform particle size (0.5e1 mm diameter). The bone powder was then loaded into a flow cell (a modified chromatography column) and, using a computer controlled pumping system, demineralized using 0.5 M hydrochloric acid, extracted with 0.1M sodium hydroxide (NaOH) to remove humic and fulvic acids, and then rinsed with weak acid (0.001M HCl). After ensuring each sample had a final pH of 3, the insoluble collagen fraction was removed from the flow cell and gelatinized by heating to 70°C for 20 h. Each gelatinized sample was cooled to room temperature, filtered through a 0.45mm glass microfiber filter with polypropylene housing, and then lyophilized. Carbon isotope stable ratios of the samples were measured by the Accelerator Mass Spectrometry laboratory at the University of Arizona, Tucson, AZ. Finally, a few specimens from Mongolia were radiocarbon dated following the bone collagen extraction procedure described in Zazzo et al. (2019)[144]. Here, AMS measurements were performed at LMC14 and LSCE using the (Artemis platform and ECHo-MICADAS facilities, respectively. For the Artemis-dated sample, CO2 was extracted and purified by combustion at the MNHN laboratory, then sealed in a glass tube. Graphitization was carried out at LMC14. Collagen samples dated by ECHo-MICADAS were combusted then reduced to graphite form at LSCE via AGE3 equipment before measurement on ECHo-MICADAS according to the procedure described in Zazzo et al. 2019[144].

Table S1 provides raw radiocarbon dating measurements (uncal. BP, Before Present), and calibrated dates, resulting from calibration in OxCal online (https://c14.arch.ox.ac.uk/oxcal/OxCal.html), using the IntCal2020 calibration curve[50]. Dates generated as part of this study are flagged ('1') on the column labelled 'ThisStudy' on Table S1, section 'Radiocarbon Dating Information'. Likewise, those previously published are flagged on the columne labelled 'PreviousStudy', with reference to the original publication reporting the date for the first time (column 'Reference', section 'Radiocarbon Dating Information'). Finally, the column 'Lab Reference' reports the official name of the radiocarbon date considered.

Section 3. Genome Analyses

Comparative panels

In this study, we generate the genomes of 124 ancient horse specimens to an average sequencing depth of 1.822-fold (0.288-10.925-fold). Their remains were recovered from across Eurasia, and, combined, help documenting the last seven millennia of horse evolution (from 5,100 BCE to 1,800 CE). We merge all them with 352 ancient and 81 modern genomes that were previously generated, the latter of which representing 40 breeds as well as the Przewalski horse[2,7,30,59–61,68,120,145–155,155–160]. This assembles 558 genomes in total, and provides the most extensive time-series ever released for a non-human species, which also includes 401 radiocarbon dates, 140 of which new (Table S1). This panel encompasses all horse lineages known to have ever-populated Eurasia, in addition to eight phylogenetic outgroup specimens, namely two *Equus ovodovi* equids[60], four donkeys[59,153], and two phylogenetically-closer horses from the Late Pleistocene North America (LP-NAMR)[61]. These outgroups facilitate the polarization of alleles as ancestral or derived: the nucleotide variant common to the three outgroup lineages is considered ancestral. Because of the paraphyly of the different outgroups considered[61], such an ancestral state can be confidently considered as the nucleotide variant carried by the most recent common ancestor (MRCA) of LP-NAMR and all Eurasian horses, *ie.* ancient and modern.

Our new genomic time-series densely covers the whole temporal range of horse domestication, starting with the site of Botai, north central Kazakhstan, which is associated with the earliest evidence for horse milking, bridling and corralling ~5,500 ya[6]. It was also selected to encompass the origins and further development of DOM2 horses, from their genetic homeland in the lower Don-Volga region ~2,200 BCE (Table S1). More specifically, it purposely over-represents Europe, the Carpathian basin and surrounding areas, prior and after ~2,200 BCE. These regions have recently regained interest, following three independent lines of research.

The first line of research relates to a new computational method, Admixtools2, attempting automatic inference of complex population graphs[4]. This method inferred ~20% introgression from ancient DOM2 horses into horses affiliated to the Corded Ware Complex (CWC; ~2,700 BCE). As this culture mainly developed from Yamnaya-related steppe pastoralists that migrated into Central Europe ~3,000 BCE, such DOM2 introgression was used to suggest that steppe pastoralists expanded into Europe together with their steppe horses[4].

The second line of research pertains to an archaeological study of pre-Yamnaya and Yamanya-related human remains, which documented a few chronic skeletal lesions tentatively associated with frequent horse riding in the Carpathian basin[5].

The third and last line of research corresponds to an ancient DNA study that proves the early arrival of humans from the steppe into the Carpathian basin, commencing from 4,500 BCE[10]. Combined, these three pieces of evidence could suggest that horse-based mobility and, thus horse domestication, occurred much earlier than previously anticipated. Our comprehensive panel was, therefore, not only designed to represent an unprecedented resource for documenting the past and present genomic diversity in horses, but also to serve as a direct test to whether or not the horse accompanied past human migrations, including those associated with the dispersal of Proto-Indo-European (PIE) languages.

Ancient DNA sequencing

DNA was extracted from horse archaeological remains and manipulated in the state-of-the-art ancient DNA facilities of the Centre for Anthropobiology and Genomics of Toulouse (CAGT, France). The procedures underlying ancient DNA extraction, USER-treatment as well as the preparation, amplification and purification of DNA libraries were detailed in[2,7,30]. DNA libraries were screened using shallow Illumina sequencing to detect those showing substantial endogenous DNA content, therefore, allowing the cost-effective characterization of ancient genomes using shotgun sequencing. Final sequencing efforts were carried out on Illumina HiSeq2500, HiSeq4000 and NovaSeq6000 (S4) instruments, using paired-end sequencing (2x76, 2x76 and 2x150 cycles), excepting for samples BotaiB_Kaz_m3228, Rus8_Rus_m855 and VEM107_UK_956, which were characterized using single-end sequencing (81 cycles, as libraries included single unique indexes *vs* three indexes, *ie.* two internal and one external, for all others). Raw sequencing reads were processed using the Paleomix pipeline (v1.2.13.2)[53], which automates adapter trimming, read demultiplexing and collapsing, mapping (and local realignment around indels) against the horse reference nuclear genome (EquCab3)[55], the horse reference mitochondrial genome (Accession nb = NC_001640)[56] and the Y-chromosomal contigs from

Felkel and colleagues[45]. PCR duplicate removal and alignment quality filtering (minimum mapping quality = 25) were also carried out as part of Paleomix.

Sequence data trimming, rescaling, and pseudo-haploidization

Ancient genomes typically include rampant fractions of nucleotide mis-incorporations at sites affected by post-mortem DNA damage (see[161], for a recent review). To improve the quality of the ancient DNA data, read alignment files were trimmed and rescaled according to the procedure described by Librado et al. (2021)[2], binning alignments according to their likelihood to contain nucleotide mis-incorporation resulting from post-mortem DNA damage, as determined from PMDtools (v0.60)[62]. Those alignments showing PMD scores not suggestive of post-mortem cytosine deamination (--upperthreshold 1) were trimmed for 5 bp at their termini, or for 10 bp after being rescaled for their base qualities at sites showing PMD scores suggestive of post-mortem cysotine deamination (--threshold 1). The latter was carried out using mapDamage2 (v2.0.8)[58], with default parameters. The resulting alignments, in both bins, were then merged into a single bam alignment file for further processing, using samtools v1.11 (using htslib 1.11)[162]. Overall, the raw median depths-of-coverage of all ancient horse genomes was 2.22-fold (min=0.17-fold, max=28.01-fold overall; or median=1,70-fold, min=0.33-fold, max=13.26-fold for those newly characterized in this study). It was reduced to 1.80-fold (min=0.13-fold; max=25.08-fold), once sequence alignments were trimmed and rescaled.

In order to prevent uneven sequencing depths from impacting downstream analyses, all 558 genomes were pseudo-haploidized, following the procedure described in the supplementary information of Librado and colleagues[1]. This procedure entails pseudo-haploidization by randomly sampling an allele from the pile of reads mapping against polymorphic nucleotide transversions, as detected through joint SNP calling across the 558 specimens (-SNP_pval 0 in ANGSD v0.917[63]). Nucleotide transitions and InDels were, thus, disregarded. To further improve calling accuracy, two new SNP filters were added before proceeding with downstream analyses. First, we identified the presence of an excess of transversions that were separated by 1 bp only (Extended Data Fig. 1a). These could represent true successive or multi-nucleotide variants, or mapping errors around nearby InDels. As the latter would artificially increase sequencing error rates, we randomly excluded one of the two adjacent nucleotide transversions, which could otherwise bias the recombination clock by distorting genetic linkage at extremely short-ranges. Second, we found that nucleotide transversions covered in less than 70% of the specimens were systematically depleted in low-frequency alleles (Extended Data Fig. 1b). This observation reflects poorer performance in discovering low-frequency variants in the presence of elevated missingness, as expected. We, thus, restricted our analyses to sites including at most 30% missingness, be polymorphic or not. Overall, our final data set retained a total of 9,099,487 high-quality nucleotide transversions spread across the 31 horse autosomes.

Using the full data set, nonetheless, we found that some of the downstream analyses were still slightly biased by uneven sequencing depths. This is due to the strategy of joint SNP calling, which is underpowered to detect singleton variants, if the carrier sample is sequenced at low depths (Extended Data Fig. 1b). To mitigate this, we downsampled all horse genomes to the median sequencing depth present in the full panel (2.02-fold), using samtools[162]. We also restricted downstream analyses to sites with less than 30% of missingness, which yield 7,092,366 nucleotide transversions (downsampled data set; Extended Data Figs. 1cd). Such a depth homogenization is not deprived of undesired effects, as it ultimately levels down data quality, and decreases our power to detect SNPs but also to identify and filter out sequencing errors. Given their respective pros and cons, we replicated every single downstream analysis on the full and the downsampled data sets. Both provided consistent conclusions, unless otherwise stated.

Relative error rates

The relative error rate for each pseudo-haploidized genome was estimated by quantifying its proportion of derived alleles, as measured from all outgroup lineages, relative to the proportion of derived alleles found in a high-quality modern genome that served as baseline reference (P5782_Ice_Modern). An excess of derived mutations in the test genome would reveal inflated error rates, relative to P5782_Ice_Modern. Genomes lacking mutations, in contrast, reflect missing derived alleles, due to low-depth sequencing or to the shorter evolutionary trajectory of ancient samples, which stopped evolving when the underlying specimen died (the latter prevented the further accumulation of mutations, in contrast to the genomes of modern samples). The

relative error rates for the full and the downsampled data set are provided in Extended Data Fig. 2. Note how a few specimens exhibit more C-to-A and G-to-T errors in the downsampled data set, arising from chemical oxidation, due to the impossibility to filter them out based on limited sequencing information. This effect reflects the unsatisfied balance between true variant discovery and error filtering.

The genetic makeup of CWC and Central European horses

Recent genomic research has revealed that ancient horse populations exhibited a marked spatial structure, characterized by strong patterns of isolation-by-distance[2]. This structure persisted until ~2,200 BCE, when the lineage of modern domestic horses (DOM2) left their original homeland, the lower Don-Volga region. Following their expansion across Eurasia, DOM2 horses ultimately replaced all other local lineages, excepting the endangered Przewalski's horses. Solid genomic analyses supported this chronology, including phylogenomic inference, Struct-f4 ancestry profiles[24], PCA and multidimensional scaling, demographic (N_e) trajectories, mobility patterns over time, as well as spatial projections of the DOM2 genetic makeup based on pre-2,200 BCE clines of genetic ancestry[2]. Assuming from zero to five migrations, OrientAGraph[163] returned a population model aligned with this timeline, which consistently placed DOM2 horses as a sister group to ancient steppe populations (C-PONT and TURG, ~3,046 and 2,723 BCE on average, respectively). Together, C-PONT, TURG and DOM2 horses formed a monophyletic clade highly isolated from other horse populations outside the steppe, including CWC horses.

That the global expansion of modern domestic horses occurred ~2,200 BCE entails deep implications for understanding past human migrations. As such, DOM2 horses expanded approximately eight centuries after humans massively spread out of the same region, with the so-called Yamnaya phenomenon, which extensively reshaped the linguistic and genetic landscape of Europeans 3,000 BCE[8,9]. Such chronologies imply that ancient human populations had capacities for fast dispersion well before horse riding, in contrast to what commonly assumed[19]. Recent research has further supported early human mobility decoupled from horse riding, by documenting human migrations from the steppe into the Carpathian basin starting 4,500 BCE[10], a temporal boundary anterior to any evidence for horse domestication.

In this context, Maier and colleagues developed Admixtools2, a computational tool that implements an optimization routine for automatically fitting admixture graphs to f_3-statistics[4]. They showcased their method' applicability by revisiting some previously-published admixture graphs, including the OrientAGraph population graphs originally reported by Librado and colleagues[2]. Using their automated procedure, they found population models with eight migration edges, in which DOM2 contributed ~20% ancestry into CWC horses (|WR|= 3.38 SE). This high proportion of steppe-related horse genetic ancestry in animals that lived ~2,700 BCE in Europe could lend credence to the notion that horses may have accompanied past human migrations out of the steppe ~3,000 BCE, despite the results from other genomic analyses indicating otherwise.

We posit that this fundamental discrepancy could be attributed to the use of biased f_3 values, occurring in the presence of population groups represented by a single pseudohaploid sample (pseudohaploid population groups)[4]. In Librado et al. (2021)[2], the population group TARP was pseudohaploid, comprised of the only Tarpan genome sequenced at the time, whereas two other groups (NEOANA and DONK) became represented by only a single pseudohaploid individual after Maier and colleagues modified the original group configurations. In an effort to address this potential bias, we extend the panel of specimens, incorporating three additional donkey specimens, two NEOANA horses, and another Tarpan into their corresponding population groups. Using AdmixTools2, we then found another graph that does not support gene-flow from DOM2-related horses into CWC horses (Extended Data Fig. 3b). Using the *qpgraph_resample_multi* function of the Admixtools2 package, we confirmed this new Admixtools2 graph as significantly better than that favored by Maier and colleagues (P < 1e-5).

To further understand the genomic makeup of European horses prior and after 2,200 BCE, we added a whole series of new analyses, including a wider diversity of population groups than those originally considered by Librado et al. (2021)[2]. These involved Neolithic horses from present-day Poland (NEOPOL), Eneolithic horses from present-day Czechia (ENEOCZE) and Romania (ENEOROM), Middle Bronze Age horses from the Carpathian basin (HUNG), and the most ancient population from the steppe in our extended dataset (NEONCAS) (Table S1). Collectively, these 14 population groups span the period from before, during and after the Yamnaya expansion, hence providing increased resolution to quantify potential genetic contributions

from the steppe into Europe. Considering the complexity of this horse dataset, we leveraged the Bayesian framework provided by AdmixtureBayes to naturally capture and summarize the uncertainty associated with graph inference. We carried out three independent runs of AdmixtureBayes, each containing 163 MCMC chains communicating in parallel, during 200 million MCMC iterations. Graphs assuming M = 13 migration events received the best posterior probabilities, and fitted remarkably the *f*-statistics calculated by AdmixTools2 (|Zscores|<3.34). Both the best AdmixtureBayes model (Extended Data Fig. 4) and the 99% consensus of the posterior distribution (Extended Data Fig. 3a) confirmed no DOM2 nor steppe-related ancestry into CWC horses.

To further characterize the CWC genetic makeup, we used qpAdm modeling[65] (version 1520, within AdmixTools 7.0.2), and rotated all steppe and European horse populations, in addition to DOM2, as potential donors of ancestry to CWC (Table S2, allsnps = NO). The CWC genomic makeup was successfully explained by a two-source mixture of populations located in close geographic proximity to the true CWC sampling location ($P = 0.109$), namely: the South ENEOCZE population form Czechia (32.4%) and the North FBPWC (Funnel Beaker/Pitted Ware horses) from Denmark (67.6%). Both parental populations predate the arrival of Yamanya steppe pastoralists into their respective regions (Table S1). All the remaining two-way models were unfeasible or explicitly rejected ($P < 0.01$), including those considering steppe or DOM2 horses as donor (left) populations. Allowing for a third population source returned a best model in which pre-Yamanya horses from Poland (Funnel Beaker, FBCPOL) also contributed ancestry to CWC horses ($P = 0.38$). Other 3-way qpAdm models were not rejected either, but returned negligible levels of steppe (<1.7%) or DOM2 (0.3%) introgression into CWC horses.

To assess the validity of the population graph obtained, we implemented a methodological framework free of *a priori* group definitions[21]. To achieve this, we used deep neural networks to predict the spatial origins of the CWC genetic makeup, according to pre-existing patterns of isolation-by-distance, which were modeled through a reference panel encompassing 148 horse specimens sampled across Eurasia. The rationale is that ancient clines of genetic ancestry provide increased statistical power to interpolate the geographic origins of a more recent query sample. We queried each CWC horse separately, assuming no missing data to prevent biases resulting from the imputation strategy implemented by Locator[21]. This crucial filtering left more than 1.2 million nucleotide transversions for the query CWC sample sequenced at lower depth, still surpassing the guidelines outlined in the original Locator publication[21]. We thus partitioned the genome of each CWC horse into 10Mb chunks, representing an intermediate window size and an excellent trade-off between informativeness and resolution to identify local ancestry tracts potentially originating from the steppe. In this regard, Maier and colleagues interpreted the ~20% DOM2 ancestry into CWC horse as possibly related to the Yamnaya expansion out of the steppe, 3,000 BCE. This is at most ~300 years (~37.5 generations) before the radiocarbon date of CWC horses (~2,700 BCE). This short timescale implies that low-recombining regions along the CWC genomes should still harbor long ancestry tracts, unbroken by recombination, if truly originating from the steppe. For every 10Mb window, we executed Locator 50 times and computed the average projections, weighting them based on their corresponding validation errors. No 10Mb-long genomic window projected into or toward the steppe. All were instead centered around the true sampling location of CWC horses, reaffirming their identity as local European lineage (Extended Data Fig. 3cd).

All the analyses presented above align with the additional evidence presented below, consistently supporting the makeup and expansion of modern domestic horses out of the steppe from ~2,200 BCE. Comprised of 558 genomes, our extensive genomic time-series allowed us to monitor this population turnover at the Carpathian and Transylvanian Basins at an unprecedented fine temporal resolution, even within the same archaeological sites (Budapest-Királyok Útja and Százhalombatta-Földvár; Table S1). In contrast to CWC horses from Central Europe, ancient horses from these basins (geographically connected to the steppe) carried significant amounts of steppe ancestry, but were not DOM2. With an average of ~54%, the steppe ancestry detected was maximized in ENEOROM horses (~4,200 BCE), and to a lesser extent of ~17% in HUNG horses (~2,200 BCE). Older specimens from eastern Austria (~3,300 BCE) and Neolithic Poland (NEOPOL, ~5,100 BCE) carried similar or even greater levels of steppe ancestry (~29% and 15% respectively), confirming natural diffusion of horse genetic ancestry from the steppe prior to any documented human migration, or to any evidence for horse domestication. This is in line with geographic clines of genetic ancestry (Extended Data Fig. 5), formed following isolation-by-distance. Regardless of their steppe ancestry, we found that all these local

lineages started to be swept away following the expansion of modern domestic horses ~2,150 BCE, although some pre-DOM2 horses persisted in the region at least for an additional century, until 2,050 BCE (Fig. 1cd).

Population structure

The extensive genomic time-series gathered in this study includes 124 newly generated genomes, of unknown genetic ancestry. We, therefore, characterized their genomic makeup using the Struct-f4 package[24]. To decrease computational costs, we grouped 272 horses previously identified as DOM2 into a single population (DOM2), including all modern breeds. The two *E. ovodovi* specimens were similarly grouped as OVO, and the four donkey specimens as DONK. This reduced the number of population groups from 558 to 283. While fixing either OVO or DONK as outgroup lineages, this led to 22,128,750 configurations of the $f_4(x, y\,;\, w, OVO|DONK)$ statistics. This number of combinations results from:

$$f_4 \text{ permutations if considering DONK as outgroup} + f_4 \text{ permutations if considering OVO as outgroup} - f_4 \text{ permutations overlapping in both datasets} =$$

$$3 \text{ x } \binom{282}{3} + 3 \text{ x } \binom{282}{3} - \frac{3}{2} \text{x} \binom{281}{2}$$

The combinatorial terms in the first and second expressions provide the number of distinct triplets given 282 non-outgroup populations. As each population can act as x, y or w within each triplet, these combinatorial terms are then multiplied by three to obtain the total number of unique f_4 permutations if fixing either DONK or OVO as outgroups. The third expression excludes redundant f_4 permutations, overlapping in both datasets. These redundant f_4 permutations place DONK as outgroup, and OVO as x, y or w, or *vice versa*.

The ~22.1 million f_4 configurations were then used to estimate the allele frequency shifts between each pair of specimens. The resulting semi-matrix of pairwise genetic distances was in turn processed through Multi-Dimensional Scaling (MDS), to obtain a three-dimensional representation of the underlying population structure (Extended Data Fig. 5). All outgroup lineages, namely the two LP-NAMR horses from North America, OVO and DONK were excluded prior to MDS visualization. The goodness of fit of the resulting MDS was 81.95% and 82.6%, for the full and downsampled data sets, respectively. These indicate that both MDS representations provide an accurate low-dimensional summary of the underlying genetic affinities.

Only horse specimens that were radiocarbon-dated as more recent than 2,150 BCE projected together with DOM2. The MDS visualizations also highlight the clines of genetic ancestry that prevailed before that time, with CWC horses clustering together with other native European specimens that lived before the Yamnaya-related migration ~3,000 BCE, including a 34,800 BCE specimen from Romania (RONPC06_Rom_m34801), as well as Late Pleistocene horses from northern France (LPNFR; Table S1). The MDS space between steppe and Central European horses was occupied by a genetic cline that represented a spatially intermediate contact zone, including eastern Europe, the Carpathian and Transylvanian Basins. Some horse specimens within this contact zone are ~5,100 BCE (NEOPOL), suggesting that this cline was already in place prior to any human migration documented from the steppe[10]. Further ancestry decomposition of the genome variation into K = 8-10 components returned sample-specific genetic profiles that corroborated the presence of steppe ancestry into NEOPOL, and also a slight, but significant, proportion into Late Pleistocene horses from France (LPNFR and LPSFR), suggesting an early wave out of the steppe refugium, possibly in the aftermath of the Last Glacial Maximum (LGM) (Extended Data Fig. 6). DOM2 and CWC horses were the most separated along the third MDS dimension, in comparison to other European horses. This is relevant because our MDS only relies on f_4 permutations, and is thus immune to sample-specific genetic drift, in contrast to classical Principal Component Analyses (PCAs). Consequently, the separation between CWC and DOM2 horses in the MDS space is most likely reflecting their mutual isolation from each other.

Horse inbreeding levels

Breeding domestic animals can involve the reproduction within close kins, otherwise called inbreeding, as a practice aimed at propagating desirable traits[37]. In order to determine when this practice was first established

in horse domestication history, we estimated inbreeding levels within DOM2 and Botai horses, using a reference-free approach, *ie.* agnostic on population allele frequencies. This approach was recently implemented in donkeys[59], and involves random sampling two reads per nucleotide transversion and individual. Sites with sequencing depths lower than 2-fold are, therefore, skipped and positions in which the two sampled reads carried the same allele are considered pseudo-homozygous. If the two reads randomly sampled supported different alleles instead, the position is considered pseudo-heterozygous. We calculated the resulting pseudo-heterozygosity in 1 cM-long genomic windows along each individual genome. Windows with a pseudo-heterozygosity lower than 0.005 were considered Runs of Homozygosity (ROHs) and the cumulated span of these ROHs provided proxies for inbreeding.

Horse populations suffering from long-term small population sizes lead to patterns of inbreeding characterized by both, short and long ROHs. Close kin mating, in contrast, is expected to exclusively yield long ROHs, as spared from recombination. As horses are known to avoid close kin mating in the wild, genomes characterized by long ROHs only (≥15 cM) are thus unexpected unless close kin mating is forced and controlled by breeders. To distinguish both scenarios, we thus categorized ROHs into these five length bins: (i) in between 1-2 cM, (ii) 2-5 cM, (iii) 5-10 cM, (iv) 10-15 cM, and (v) above 15 cM.

While Botai horses were found the least inbred (Fig. 2c), elevated levels of inbreeding were found in almost all wild populations before 2,200 BCE. This included sporadic specimens from NEOBOR (Central Asian steppe), NEOANA (Anatolia), and especially ENEOROM, FBPWC, and CWC, all from Europe. Their form of inbreeding, however, was mostly present in the form of short ROHs (≤2 cM), in line with extreme habitat fragmentation documented at the beginning of the Holocene, a time period in which horse population only survive as relict, isolated populations. In contrast to these naturally-inbred populations, low inbreeding levels were found within the native range of ancient DOM2 horses, supporting that the steppe was a preferred habitat (refugium) hosting larger horse populations[164]. Carrying a significant proportion of ROHs exceeding 15 cM, the earliest instances of close kin mating amongst ancient DOM2 horses were associated with Sintashta archaeological contexts (Fig. 2c), namely RN03_Rus_m1851 (12.6% of its genome in ROHs ≥ 15 cM), UR17x47_Rus_m1856 (6.2%), and UR17x31_Rus_m1845 (5.1%). This finding demonstrates that breeding within close genealogical kins started with the earliest stages of DOM2 domestication, ~2,200 BCE, and not earlier.

Reconstruction of demographic trajectories

Previous work suggested that Botai people may have developed horse husbandry to secure a food resource that was declining in the wild, and could not sustain the subsistence need of a settled population[7]. From an evolutionary standpoint, this hypothesis implies that the horse population size decreased over time, at least from the time period motivating husbandry.

To assess such predictions, we pseudo-haploidized the genomes of 34 horses excavated at Botai, following the methodology described above. To avoid over-representing pairs of related individuals, we retained the specimen that was sequenced to deeper coverage in each pair previously identified as related (*ie.* Botai1 and Botai2018x26), and disregarded the other pair member (*ie.* Botai4 and Botai2018x30, respectively). We also excluded individuals with a missingness superior to 10%, retaining 28 high-quality Botai horse genomes in total. Their demographic dynamics was reconstructed using GONE[26], a method exploiting patterns of linkage disequilibrium (LD) to infer N_e population trajectories during the 200 generations leading to the population of interest (average age of the radiocarbon-dated Botai horses ~ 3,342 BCE; Table S1). GONE was run with default parameters, except for the option PHASE = 0 (for pseudo-haploid data). We also removed chromosomes 7, 11, 12 and 20, given our concerns on their genetic map (see below). A total of 500 replicates were run to assess confidence intervals for N_e estimates. The resulting demographic trajectory revealed N_e increments commencing ~75 generations prior to the age of Botai specimens, and peaking at N_e = 16,000 (8,000 reproductive individuals) ~55 generations later. From that time period onwards, the population effective size continuously decreased down to N_e = 12,000 (6,000 reproductive individuals). According to the simulations conducted by Santiago and colleagues[26], this trajectory could theoretically reflect the emergence of geographic structure in the population investigated, or the introgression from an external population. However, no obvious genomic differences structured the Botai horse population, and our analyses followed the GONE developer recommendations to restrict the maximum genetic distance analyzed to 0.05 cM in order to ignore those long

LD blocks originating from recent gene flow, which can introduce spurious N_e increments following sporadic admixture pulses. The N_e trajectory inferred is, thus, most likely reflective of a true population demographic decline, with the expansion phase mirroring paleoclimatic reconstructions suggesting a short period of increased humidity and a more favorable habitat for horses in the region[38].

We next evaluated the demographic trajectory along the 200 generations preceding the earliest high-quality DOM2 horses in our data set (n = 24; average age ~1,864 BCE; Table S1). We found a demographic profile dramatically different than that inferred for Botai horses, characterized by a long and massive population bottleneck at a time immediately predating the DOM2 expansion across Eurasia (Fig. 2a). An almost identical demographic trajectory was recovered when conditioning the analyses on 14 DOM2 horses affiliated to the Sintashta culture (Extended Data Fig. 7a). Although the methodology used is less reliable for exploring older changes in N_e, no other bottlenecks were found in the previous 400 generations, which strongly suggests that the recent bottleneck detected was unique and related to the formation of DOM2 horses. This possibly involved selection at the *GSDMC* and *ZFPM1* genes, given that the most common allele in DOM2 horses was not present in more ancient specimens from the region, such as C-PONT and TURG[2]. This finding aligns with close kin mating at some of the Sintashta horses examined.

Section 4. Measuring temporal variations in the horse generation time

Motivation

Based on a more limited number of samples, previous work reported an expected negative correlation between the number of derived mutations present in a given horse genome and the radiocarbon age of the underlying sample[2]. This correlation is only expected if sequencing errors are limited, and likely reflects that the genome of more ancient samples show a deficit of true mutations due to their shorter evolutionary time spans, relative to more recent specimens (*ie.* have experienced a lack of generations to accumulate mutations). If confirmed, temporal accumulation of mutations, relative to an outgroup, represents a direct way to measure the horse mutation clock at the genome-wide scale. If non-linear with time, this mutational clock would reflect time periods in which generation times were shortened or extended, accelerating or decelerating the accumulation of mutations in absolute time-scales (radiocarbon years).

In contrast to temporal variations in the mutation rate, shifts in generation times also affect the recombination clock, since recombination events are also expected to have accumulated at an approximately constant rate per generation. With shorter (longer) generation times, the genetic linkage between adjacent mutations would be maintained for shorter (longer) time periods (in absolute time scales). In order to identify time periods in which past horse herders may have accelerated the horse generation time (*eg.* for increasing the reproductive productivity, hence, their capacity to sustain possibly growing animal demands), we devised statistical methods aimed at measuring changes in the horse generation times, from both mutation and recombination clocks.

Mutation clock

It has been previously established that the proportion of derived mutations in pseudo-haploidized genomes is insensitive to their underlying demographic history (*ie.* the changes in their effective population size; Extended Data Fig. 8a)[29]. This finding is corroborated through the coalescent simulations described in the following sections. To obtain a mutational clock, we measured the proportion of derived mutations present in the genome of a given sample *i*, as follows:

$$d_i = \frac{\#derived\ mutations_i}{\#sites_i}$$

The numerator was calculated from the panel of jointly-called SNPs (*ie.* with -SNP_pval 0 in ANGSD v0.917[63]), whereas the denominator accounted for all positions covered in sample *i*, regardless whether SNPs or not (*ie.* without -SNP_pval 0). All remaining QC filters were equivalent for both the numerator and denominator (*eg.* 30% missingness at best in the full data set; see above). The number of generations required to accumulate such a d_i proportion of derived mutations can be directly learnt from the mutation rate (μ, mutations per site per generation), as follows:

$$g_i = \frac{d_i}{\mu}$$

We assumed the rate of $\mu = 7.242\text{x}10^{-9}$ mutations per generation, following the work by Orlando and colleagues[29]. Considering a realistic transition:transversion rate of 2.2 implies that one in every 3.2 mutations corresponds to a transversion. We thus scaled the transversion rate accordingly, to $\mu = 2.22625\times10^{-9}$ nucleotide transversions per generation and site. By relying on nucleotide transversions only, we eradicated the impact of uneven post-mortem DNA damage across our data set. It also allowed for a direct comparison of ancient and modern data, within single analyses.

Applying the calculation above to the full data set, horses were inferred to have diverged from LP-NAMR horses some $g \approx$ 92,000-115,000 generations ago, which corresponds to 736,000-920,000 years ago assuming mean generation times of 8 years (Extended Data Fig. 8b). This estimate matches independent inference based on G-PhoCS reconstructions[165] and relying on high-quality genomes[61], which broadly validates our SNP calling strategy, despite the more moderate genome coverage achieved in the present study. Standard

errors were calculated using chromosome-scale jackknifing (*ie*. leaving out one chromosome for each replicate), but were not displayed in Extended Data Fig. 8b to avoid overplotting issues. Using the downsampled data set, horses were inferred to have diverged from LP-NAMR $g \approx$ 100,000-122,000 generations ago (Extended Data Fig. 8c), which, although slightly inflated, largely overlaps the estimate based on the full data set. We interpret this slight discrepancy as reflective that the downsampled dataset suffers from more elevated error rates, which introduce spurious mutational counts, leading to deeper divergence times.

Recombination clock

Recombination operates each generation, breaking up physical linkage between variants. The probability that recombination never occurred between two SNPs located m Morgans away from each other, after t generations of evolution from the MRCA, is:

$$P_{linked} = (1-m)^t$$

This equation is simple to interpret, as it indicates that with increasing genetic distances (greater m), and/or number of generations evolved (greater t), the probability to remain fully linked decays nearly exponentially. Conversely, we have:

$$P_{unlinked} = 1-(1-m)^t$$

By definition, fully linked SNPs retain the same genealogical history, hence, the same time to the MRCA (tMRCA). This identity in tMRCA implies that both SNPs have equal expectations to carry a derived or ancestral allele, in contrast to SNP pairs located in separate haplotype blocks. This rationale sets the scene for deriving measurements of generation times from the recombination clock (Extended Data Fig. 9a). More specifically, the theoretical expectation to find two pairs of SNPs separated by m Morgans, and carrying both a derived allele in sample i, is given by:

$$E(D_{mi}) = \frac{t_{Li}}{T_L}\left[(1-m)^{xt_i}\frac{t_{Li}}{T_L} + (1-(1-m)^{xt_i})\frac{t_{Ri}}{T_R}\right]$$

where t_{Li} depicts the tMRCA at the left SNP in sample i, and T_L is the total branch length of the whole genealogy for that SNP. Their ratio t_{Li}/T_L is, thus, the probability that sample i carries a derived allele at the left SNP (Extended Data Fig. 9b). If the right SNP remained linked at the time when the second mutation appeared (xt), its probability to carry also a derived allele is again t_{Li}/T_L (otherwise, its time to the tMRCA changes to t_{Ri}/T_R). Rearranging the equation above, we have:

$$E(D_{mi}) = \frac{t_{Li}}{T_L}\left[(1-m)^{xt_i}\left|\frac{t_{Li}}{T_L} - \frac{t_{Ri}}{T_R}\right| + \frac{t_{Ri}}{T_R}\right]$$

where the subtraction

$$\left|\frac{t_{Li}}{T_L} - \frac{t_{Ri}}{T_R}\right|$$

represents the standard deviation of the tMRCA along the genome i, relative to the total length of the genealogy. Because measures of data dispersal cannot be negative, we calculate its absolute value. Note that measuring the probability of linkage (ie. $(1\text{-m})^{xti}$) from the left to the right SNP is arbitrary, and conceptually could be equally reversed from the right to the left SNP, leading instead to:

$$E(D_{mi}) = \frac{t_{Ri}}{T_R}\left[(1-m)^{xt_i}\left|\frac{t_{Ri}}{T_R} - \frac{t_{Li}}{T_L}\right| + \frac{t_{Li}}{T_L}\right]$$

This conceptual arbitrariness mathematically requires expressing the standard deviation in absolute terms. Interestingly, the standard coalescent predicts that such standard deviation is simply equivalent to t_i/T, where t_i and T are the corresponding averages along the genome. The presence of population size changes (N_e) and recombination, however, invalidate the applicability of the standard coalescent, meaning that this standard deviation becomes instead proportional to an unknown constant p_i, which captures both N_e fluctuations experienced by sample i as well as the average length of its haplotype blocks:

$$E(D_{mi}) = \frac{t_{Li}}{T_L}\left[(1-m)^{xt_i}\frac{t_i p_i}{T} + \frac{t_{Ri}}{T_R}\right]$$

By considering that we are working on expectations ($t_{Li} = t_{Ri}$ and $T_L = T_R$), the above expression can be now reformulated, without much loss of generality as:

$$E(D_{mi}) = \frac{t_i}{T}\left[(1-m)^{xt_i}\frac{t_i p_i}{T} + \frac{t_i}{T}\right] = \frac{t_i^2}{T^2}\left[(1-m)^{xt_i}p_i + 1\right]$$

The product xt_i determining the expected time required for two mutations to appear, however, remains thus far unknown, dependent on x. Solving it analytically requires to integrate over all possible times of mutation occurrence, at both the left and right SNPs, as described below:

$$E(xt_i) = \int_{L=1}^{t_i} \frac{\mu(1-\mu)^{L-1}}{1-(1-\mu)^{t_i}} \int_{R=1}^{t_i} \frac{\mu(1-\mu)^{R-1}}{1-(1-\mu)^{t_i}} \max(L,R)\, dRdL \approx \int_{L=1}^{t_i}\int_{R=1}^{t_i} \max(L,R) dRdL \approx \frac{2t_i}{3}$$

Replacing xt_i into previous equations, we finally obtain:

$$E(D_{mi}) = \frac{t_i^2}{T^2}\left[(1-m)^{\frac{2t_i}{3}}p_i + 1\right]$$

Because the fraction of SNP pairs carrying both derived mutations (D_{mi}) may depend on the underlying sequencing depth and error rate, we designed a more robust statistics, r_{mi}, which is obtained by normalizing $E(D_{mi})$ by the proportion of derived mutations detected within each sample i:

$$E(r_{mi}) = \frac{E(D_{mi})}{E(d_i)} = \frac{\frac{t_i^2}{T^2}\left[(1-m)^{\frac{2t_i}{3}}p_i + 1\right]}{\frac{t_i}{T}} = \frac{t_i}{T}\left[(1-m)^{\frac{2t_i}{3}}p_i + 1\right]$$

Note how r_{mi} does not rely on the mutation rate, but on the recombination rate expressed as the genetic distance between SNPs (m) as the sole source of external information (recombination clock).

For calculating m, we linearly interpolated the genetic position of each SNP from the closest markers present in the EquCab3 genetic map previously characterized by Beeson and colleagues[66]. The horse genetic map currently available, however, is not devoid of problems, for example owing to local mis-assemblies (*eg.* at multicopy olfactory receptors and immune gene families present on chromosomes 12 and 20), but also to unaccounted structural variation (*eg.* chromosome 7 harbors the largest inversion to the donkey[166]), and the presence of a new immature centromere in chromosome 11[167]. To mitigate their possible impact, we thus removed chromosomes 7, 11, 12, and 20 and considered only the remaining 27 autosomes. Applying this filtering strategy, we calculated how r_{mi} decays with genetic distance from the sequencing data of each specimen. Operationally, this entailed computing the frequency of SNP pairs at m Morgans carrying both a derived allele, standardized by the proportion of derived mutations carried by that sample.

LD decay curves, however, lose evolutionary information very rapidly as the number of generations increases. To alleviate this, we implemented a hierarchical approach, through two least square optimization procedures based on the *optim* function of the R programming language. Mathematical derivatives for the parameters p_i, t_i, and T were provided to L-BFGS-B, in the form of a gradient function, to ensure faster and more accurate convergence. The first routine searched for the p_i, t_i, and T values that best fit the oldest specimen in our data set. This was identified as R17x2_Rus_INF, a sample showing an infinite radiocarbon date, and representing the ancient horse most deprived of derived mutations (Extended Data Fig. 2). As the T parameter is common to all samples (total length of the genealogy), we effectively captured ancient LD information, which was otherwise lost in more recent specimens. The T parameter was then fixed for the second optimization step, which is aimed at estimating sample-specific p_i and t_i values. The corresponding results, for the full and downsampled data sets are shown in Extended Data Fig. 9c and Fig. 3a, respectively. Note that the estimator based on the recombination clock measures the number of generations that evolved from the ingroups' MRCA (Extended Data Fig. 9 and Fig. 3a), while the estimator based on the mutation clock estimates the number of generations elapsed from the outgroups (Extended Data Fig. 8). This difference in scaling does not impact our conclusions, since both estimators correlate almost linearly (Pearson correlation; r = 0.996 and 0.957 for the full and downsampled data sets, respectively; P < 2.2e-16).

Validation using coalescent simulations

To validate both molecular clocks, and their capacity to retrieve genuine generation time estimates in the face of confounding effects such as (1) N_e changes, (2) inbreeding and (3) data pseudo-haploidization, we conducted serial coalescent simulations using fastsimcoal v2.702[67]. For each individual, we simulated 31 chromosomes of 75 Mb each, evolving under recombination and mutation rates of 10^{-8} and $2.3\text{x}\times10^{-8}$ events per bp and generation, respectively. We sampled 20 chromosomes every 100 generations, starting 900 generations ago, to roughly span the full time period of horse domestication. The 20 chromosomes were then randomly paired, generating diploid individuals under random mating. For the most recent time period, we paired 10 of the sampled chromosomes with themselves, thereby creating fully inbred individuals. All of them were subsequently pseudo-haploidized, to mimic the impact of the data processing procedure performed on the empirical data. A total of 10 demographic scenarios were contemplated (Extended Data Fig. 10a), including:

- Scenarios A-D; Constant population sizes (N_e = 1,000, 5,000, 10,000 and 20,000)
- Scenarios E-F; Population contractions (N_e = 10,000 dropping to 1,000 either 450 or 50 generations ago)
- Scenarios G-H; Population expansions (N_e = 1,000 increasing to 10,000 either 450 or 50 generations ago)
- Scenario I; A population expansion contracting again in more recent times (N_e = 1,000 increasing to 10,000 at 450 generations ago, and declining back to N_e = 1,000 some 50 generations ago)
- Scenario J; A population contraction followed by subsequent recovery (N_e = 10,000 dropping to 1,000 at 450 generations ago, and fully recovering 50 generations ago)

Simulations confirmed both estimators as insensitive to the population demographic history, inbreeding and pseudo-haplodization (Extended Data Figs. 10bc). Both estimators, indeed, correlated with sampling age almost perfectly (Pearson correlation; r = 0.999; p-value < 2.2e-16), as intended for parameters aimed at summarizing molecular clocks, and nothing else. Limited differences were found between the ten specimens sampled within each time. Averaged over all scenarios, the root mean squared error (RMSE) around the true value was as small as 13.276 and 23.134 generations for the mutation and recombination clocks, respectively. Combined, our simulation results demonstrate that both molecular clocks calculated in this study inform on the number of generations accumulated for each particular sample, in a manner that is insensitive to demographic changes and inbreeding.

Filtering out horse specimens unsuitable for regression analyses

Because alleles are polarized as ancestral or derived based on outgroup lineages, DNA introgression from these external lineages may bring genomic runs of ancestral alleles into the ingroup samples. These not only reduce the number of derived mutations present in recipient specimens, but also increase their LD, confounding both the mutation and recombination clocks. Theoretically, these runs of ancestral alleles could be identified and subsequently masked by means of Hidden Markov Models (HMMs), but the statistical power of this technique (or similar techniques) diminishes if the true donor population is unsampled and gene flow occurred thousands of generations ago (tracts of ancestral alleles are exponentially shortened by recombination every generation, until becoming unrecognizable). This is likely the case in our dataset, with AdmixtureBayes population graphs consistently pointing to ancient introgression from basal populations into NEOANA, LPSFR and ELEN (Extended Data Fig. 4).

Notably, the Struct-f4 profiles of these populations share an ancestry component, colored in purple, that is dominant in IBE (Fig. 1b and Extended Data Fig. 6). Previous research determined that IBE indeed carries genetic ancestry from basal population(s), well reflected by an extremely divergent Y haplogroup[2,30]. To a lesser extent, this ancestry component colored in purple is also present into Vert311, NEOANA and Przewalski's horses. Interestingly, Struct-f4 estimated that this purple-colored component is the closest to that maximized by LP-NAMR horses, colored in yellow (difference in allele frequencies = 0.0439 *vs.* ≥ 0.0476 to the remaining components). This suggests increased affinities to horse populations from North America, albeit it remains unclear whether this purple-colored component represents one or more unsampled populations, as well as the demographic history that shaped such intriguing genetic affinities. Solving this question will likely require more extensive sequencing of ancient genomes from North American horse populations.

To avoid biasing our generation time estimates due to DNA introgression from outgroup populations, we excluded the IBE, LPSFR, ELEN, and Vert311 population groups, as these contained the greatest fraction of this purple-colored genetic ancestry component. We also filtered out specimens without an explicit radiocarbon date, except Botai horses due to their less ambiguous archaeological context, which provides a date approximation nearly as precise as radiocarbon dating. In total, this filtering retained 483 data points (out of 550 horses) for the regression analyses described below.

Temporal shifts in generation times

Assuming constant generation intervals, the number of generations evolved since divergence from the outgroup (mutational clock), or from the ingroup's MRCA (recombination clock), should accumulate linearly every year. This predicts a linear relation between the molecular clock estimates and the radiocarbon age of the samples investigated. The horse generation time, however, may have not remained constant, but shifted following important life-style transitions, such as climate changes (*eg.* Last Glacial Maximum; 26-19 ky BP, Before Present, ie. ~24-17 millennia BCE), and, more importantly, domestication itself. Early breeders may, indeed, have shortened (*ie.* accelerated) the average reproductive life-span of horses to rapidly multiply this revolutionizing resource, which suddenly expanded beyond its native homeland ~2,200 BCE. To test for temporal changes in generation times, we used the *mgcv* package of the R programming language, by fitting the following Generalized Additive (regression) Model (GAM) to the data:

$$Number\ of\ generations\ accumulated = P_i + \sum_{j=1}^{5} MDS_{ij} + 14C_i + \varepsilon$$

Standard errors were calculated for the independent variable, by jackknifing (leaving one chromosome out at a time), and the inverse of the resulting variance was then used as weight in the above regression model. P_i controls for the impact of uneven sequencing depths, as it approximates the probability that a derived mutation remains unsequenced in sample *i*. Because our analyses skip missing data, derived mutations are always sequenced if present in homozygous state, but they may remain unsequenced if present in the heterozygosity state. In the latter case, there is a significant chance that all sequencing reads could eventually originate from the chromosome carrying the ancestral allele. The probability of such outcome decreases with greater sequencing depths, by $(1/2)^{depth\_i}$, where $depth_i$ represents the average depth of sample *i*. Multiplied by its corresponding heterozygosity rate (h_i), this provides the proportion of missing derived mutations in sample *i*:

$$P_i = h_i * \left(1/2\right)^{depth_i}$$

The heterozygosity rate per sample *i* is approximated following the procedure underlying the inbreeding calculations, ie. by randomly sampling two reads per SNP (otherwise the site is skipped), to then count how often these two reads support the presence of alternative alleles. In the downsampled dataset, the average P_i value across all samples was 0.0056, and ranged from 0.0022 to 0.0110.

The five MDS_j variables in the regression model represent the first five coordinates of the MDS analysis described above (Extended Data Fig. 5). This is so as different population groups, such as Botai and Central European horses for example, could have experienced distinctive generation times following their divergence, owing to niche or biological specificities. If so, this could lead to an important difference in the accumulated number of generations along both evolutionary branches, even for specimens showing similar radiocarbon ages. These MDS coordinates, therefore, are set to effectively control for the underlying population structure.

The importance of accounting for population structure is well exemplified by ~2,200 BCE HUNG horses, characterized by reduced generation time estimates based on the raw recombination clock (Fig. 3a), and by an ancestry component colored in dark brown (Fig. 1b). This brown-colored ancestry component is dominant across all Central European populations and, remarkably, all these populations show similarly low generation time estimates. This suggests that the whole lineage leading to Central European horses experienced larger generation intervals, possibly due less favorable niche specificities (the steppe was likely a more favorable habitat compared to Central European woodlands, where this brown-colored ancestry was dominant, and from where it possibly diffused in moderate proportions to the Hungarian grasslands). Failure to account for population structure, therefore, would result in GAM models indicating sharp accelerations in generation times, from HUNG (~2,200 BCE) to DOM2 horses (<2,200 BCE), while in fact much of this acceleration reflects lineage-specific peculiarities following their divergence.

An even more informative example is provided by TURG horses (2,800 BCE), which show elevated generation time estimates based on the raw recombination clock and the downsampled dataset (Extended Data Fig. 7c). Located toward the Central Asian steppe, however, TURG horses carry more Botai-related ancestry than their CPONT counterparts, in line with the geographic clines of genetic ancestry existing prior to the spread of DOM2 horses (Extended Data Figs. 5 and 6). This history of admixture may have brought Botai-related DNA tracts characterized by accelerated generation times into the genomes of TURG horses, especially considering that Botai (~3,500 BCE) indeed exhibit accelerated generation times compared to contemporaneous populations from the region (Extended Data Fig. 7d). In this regard, the only valid qpAdm model identifies TURG horses as a three-way mixture of NEONCAS (0.721±0.105), DOM2 (0.121±0.082) and BOTAI (0.157±0.027) horses, and thus excludes TERSEK, Novoilinka, NEOBOR, BORL, PRZW and Aleksandrovskoe horses as potential sources of genetic ancestry (P = 0.223; Table S2). This rules out that TURG horses entirely descended from NEONCAS horses from the region, but depicts their genomic profile as shaped by continuous gene flow from neighboring locations, with at least one of these neighboring populations (BOTAI) characterized by accelerated generation times before the ~2,800 BCE age of TURG horses.

Three independent analyses support Botai introgression as the most prominent factor driving the apparently accelerated generation times in TURG horses. First, their raw generation time estimates based on the recombination clock (*ie.* the number of generations accumulated from the Most Recent Common Ancestor to all Eurasian horses in our dataset), and their fraction of green-colored ancestry, characteristic of Botai horses are nearly linearly correlated (Pearson correlation; $r^2 = 0.9847$; *p*-value = 0.02243; Fig. 1b). Second, we employed the HMM implemented in admixfrog (https://github.com/BenjaminPeter/admixfrog) to model the genomic variation along the TURG horse genomes as descending from early DOM2 and/or BOTAI populations. NEONCAS was not included as source population because our internal evaluations revealed highly volatile estimates otherwise, due to largely overlapping genetic ancestries between DOM2 and NEONCAS, and between BOTAI and NEONCAS. This uncertainty is also reflected by the large standard errors of the corresponding 3-way qpAdm model (see above and Table S2). As representatives of both parental populations, we selected the same 24 and 28 high-quality specimens used for GONE inference. Despite the two-donor

admixture model being admittedly oversimplistic, this analysis allowed us to mask genomic tracts enriched for Botai ancestry (Extended Data Fig. 7b). We then re-estimated the raw generation times for each TURG horse, and confirmed these to be considerably reduced (Extended Data Fig. 7c). Third, after effectively correcting for its distinctive ancestry profile (MDS_j), our GAM regression model remarkably predicts that the acceleration of generation times did not peak at the time of TURG horses (Fig. 3b). Instead, their elevated generation times are mostly explained by their distinctive ancestry profile, characterized by introgression from Botai specimens. Together, these analyses support that the green-colored ancestry component carried by TURG horses inflated their raw generation estimates, strongly suggesting accelerated generation times at Botai.

Lastly, the variables ${}^{14}C_i$ represent the mid-point of the radiocarbon range obtained for sample *i*, and was modeled either as linearly related to the number of generation evolved, or following cubic splines through the s() function of the *mgcv* package, using the REML method to estimate the best number of knots. Interestingly, the latter alternative provided significantly better fits (adjusted $R^2 = 0.803$ for the linear vs. 0.894 for the GAM regression; ANOVA $P < 2.2e^{-16}$). This offers statistical support for temporal changes in the horse generation time over the last 44,495 years.

To pinpoint when such shifts in generation time occurred, we next estimated the derivative of the ${}^{14}C$ regression term, using the derivative() function of the *gratia* package, with time bins of 1,000 years. Data density is scarce in the deep past, with only five radiocarbon-dated samples available prior to 28,000 BCE. After that time, a group of outlier horses from Northern France (LPNFR) show less-than-expected generations of evolution. Although no introgression from outgroup lineages is detected in their genomes, the LPNFR intermediate geographic location, in between Vert311 and LPSFR, could suggest the presence of small amounts of undetectable outgroup introgression, hence lowering their generation time estimates (see '*Filtering out horse specimens unsuitable for regression analyses*'). Because these LPNFR horses impose a lower ${}^{14}C$ slope prior to their date, we first opted for a conservative approach and estimated the mean generation time of horses over the last 20,000 only. The former time period approximately delimits the end of the Last Glacial Maximum (~24,000-17,000 BCE), and provides a mean horse generation time of 9.5 years. Restricting this analysis to the last 15,000 years period excludes the potentially misleading impact of LPNFR horses and returns a mean horse generation time of 7.4 years (Fig. 3b). Both align with the ~7-12 years commonly assumed for horses, including 12 for feral Przewalski's horses[98]. As illustrated in Fig. 3bc, this mathematical analysis also confirmed that DOM2 horses experienced short episodes in which generation times were dramatically accelerated following industrial breeding, up to a minimum of ~4.1 years per generation. This aligns with data from the most recent pedigree records[45]. Interestingly, our analyses reveal that horses underwent a comparable acceleration of generation times 2,400-2,200 BCE and not earlier, as CPONT (3,100 BCE) and TURG horses (2,800 BCE) from the steppe had evolved significantly less generations. Likewise, horses excavated at Botai were found to have accumulated more generations than both pre-existing wild populations and all other contemporaneous horse populations from the same genetic lineage and region (Extended Data Fig. 7d). Notably, the peak identified ~3,500 BCE, pinpointing another acceleration of generation times, disappears when excluding Botai and their direct descendants Borly horses from the analysis with derivatives (Fig. 3c). This indicates that Botai horses, and no other steppe-related horse populations, explain the signal of accelerated generation times in the mid-fourth millennium BCE. This may add to the multifaceted evidence supporting their domestic status, despite ongoing debates (Fig. 3 and Extended Data Fig. 7cd).

Supplementary Reference List

69. Motz-Linhart, R., Karem, M. A. E., Lauermann, E. & Rosner, W. *Keltische Festmähler und italische Rinder: Die tierischen Überreste aus dem 'Großen Heiligtum' der latènezeitlichen Siedlung Roseldorf.* (St. Pölten, 2013).
70. Richard M. Thomas. Fit for a king? A post-medieval stable and horse bone assemblage from Bradgate Park, Leicestershire. *Transactions of the Leicestershire Archaeological and Historical Society* (2023).
71. Dizdar, M., Rapan Papeša, A. & Rimpf, A. Results of the rescue excavation on the Late Avar cemetery on the site Šarengrad – Klopare. *Annales Instituti Archaeologici* **XIII**, 9–18 (2017).
72. Rapan Papeša, A., Rimpf, A. & Dizdar, M. Results of the rescue excavation on the Late Avar cemetery on the site Šarengrad – Klopare in 2017. *Annales Instituti Archaeologici* **XIV**, 13–19 (2018).
73. Majnarić - Pandžić, N. & Pandžić, N. O pojavi novih tipova konjske opreme iz završnog starijeg željeznog doba u istočnoj Hrvatskoj. *Opvscvla archaeologica* **23–24**, 27–38 (1999).
74. Rying, B. *Nordsjælland : med omliggende øer.* (Gyldendal, 1972).
75. Klassen, L. *et al.* Ginnerup Revisited. New Excavations at a Key Neolithic Site on Djursland, Denmark. *Journal of Neolithic Archaeology* 35–65 (2023) doi:10.12766/jna.2023.2.
76. Klassen, L. *The Pitted Ware Culture on Djursland: Supra-Regional Significance and Contacts in the Middle Neolithic of Southern Scandinavia.* (Aarhus Universitetsforlag, 2020).
77. Ferdinand, J. & K. Jernalderofferfund i Valmose ved Rislev. *Kuml* **11**, 47–90 (1961).
78. Foucras, S. *et al. Sépultures de Chevaux Devant Gergovie. Archéozoologie Des Rituels Gaulois.* (2019).
79. Lepetz, S. *et al.* Historical management of equine resources in France from the Iron Age to the Modern Period. *Journal of Archaeological Science: Reports* **40**, 103250 (2021).
80. Gailledrat, E. *et al.* Victorious and defeated: Warfare, rituals and funerary practices at the end of the 3rd century BC in Pech Maho (Aude, France). *Ancient West and East* **16**, 113 (2017).
81. Pirling, R. & Siepen, M. *Die Funde aus den römischen Gräbern von Krefeld-Gellep: Katalog der Gräber 6348-6361.* (Franz Steiner Verlag, 2006).
82. Schletter, H.-P. Gräberfeld, Schlachtfeld, 'vicus' – 1300 Jahre niederrheinische Kulturlandschaft in Krefeld-Gellep. *Archäologie im Rheinland* 80–82 (2017) doi:10.11588/air.2017.0.77468.
83. Günter Nobis. *Zur Frage römerzeitlicher Hauspferde in Zentraleuropa.* (1972).
84. Schmidt, B. *Die Späte Völkerwanderungszeit in Mitteldeutschland: Katalog (Nord-Und Ostteil).* (Deutscher Verlag der Wissenschaften, 1976).
85. MULLER, H. H. Zur Kenntnis der Haustieres der Volkerwanderungszeit im Mittelelbe-Saale-Gebiet. *Zur Kenntnis der Haustieres der Volkerwanderungszeit im Mittelelbe-Saale-Gebiet* **14**, 99–119 (1980).
86. Máthé, M. S. Bronze Age tells in the Berettyó valley. *KOVÁCS/STANCZIK* 27–122 (1988).
87. Staniuk, R. EARLY AND MIDDLE BRONZE AGE CHRONOLOGY OF THE CARPATHIAN BASIN REVISITED: QUESTIONS ANSWERED OR PERSISTENT CHALLENGES? *Radiocarbon* **63**, 1525–1546 (2021).
88. Duffy, P. R., Paja, L., Parditka, G. M. & Giblin, J. I. Modelling mortuary populations at local and regional levels. *Journal of Anthropological Archaeology* **53**, 240–261 (2019).
89. Depaermentier, M. L. C., Kempf, M., Bánffy, E. & Alt, K. W. Tracing mobility patterns through the 6th-5th millennia BC in the Carpathian Basin with strontium and oxygen stable isotope analyses. *PLOS ONE* **15**, e0242745 (2020).
90. Dani, J. Fortified tell settlements from the Middle Bronze Age in the Hungarian reach of the Berettyó Valley. *JAEGER, M,. CZEBRESZUK, J. & P. FISCHL, K.(eds.): Enclosed Space—Open Society. Contact and Exchange in the Contextof Bronze Age Fortified Settlements in Central Europe. SAO/SPEŚ* **9**, 27–37 (2012).
91. Csippán, P. Cultural Change and Animal Keeping: Case study of a Neolithic, Copper Age and Bronze Age Site near Budapest, Hungary. *Transition to the Bronze Age. Interregional Interaction and Socio-Cultural Change in the Third Millennium BC Carpathian Basin and Neighbouring Regions. Budapest* 319–338 (2013).
92. Bökönyi, S. Animal remains from Bronze Age tells in the Berettyó valley. *Bronze Age tell settlements of the Great Hungarian Plain* **1**, 123–135 (1988).
93. Vicze M. & Sørensen M. L. *Living in a Tell: Memory and Abandonment.* (2023).

94. Jaeger, M. & Kulcsár, G. Kakucs-Balla-domb. A case study in the absolute and relative chronology of the Vatya culture. *Acta Archaeologica Academiae Scientiarum Hungaricae* **64**, 289–320 (2013).
95. Vretemark, M. & Sten, S. *Animal Bones from the Bronze Age Tell Settlement of Százhalombatta-Földvár in Hungary.* (Matrica Museum, 2020).
96. Hüttel, H.-G. *Bronzezeitliche Trensen in Mittel-Und Osteuropa: Grundzüge Ihrer Entwicklung.* vol. 12 (CH Beck, 1981).
97. Boroffka, N. Bronze-und früheisenzeitliche Geweihtrensenknebel aus Rumänien und ihre Beziehungen: alte Funde aus dem Museum für Geschichte Aiud, Teil II. *Eurasia Antiqua* **4**, 81–135 (1998).
98. Bándi, G. Die frage der riementeiler des Mittelbronzezeitlichen pferdegeschirrs im Karpatenbeckens. *Archaeologiai Értesít\Ho* **90**, 46–60 (1963).
99. Szeverényi, V., Hajdu, T., Marcsik, A. & Kiss, V. Sacrifice, warfare, or burial? Middle Bronze Age" mass graves" from Érd and Makó, Hungary. (2020).
100. Giblin, J. *et al.* Bronze Age burials from the Carpathian Basin: new isotope results. in *AA 84th Annual Meeting, Albuquerque, New Mexico* 10–14 (2019).
101. Abedi, A. IRANIAN AZERBAIJAN PATHWAY FROM THE ZAGROS TO THE CAUCASUS, ANATOLIA AND NORTHERN MESOPOTAMIA: DAVA GÖZ, A NEW NEOLITHIC AND CHALCOLITHIC SITE IN NW IRAN. *Mediterranean Archaeology & Archaeometry* **17**, (2017).
102. Abedi, A., Varoutsikos, B. & Chataigner, C. Provenance of obsidian artifacts from the Chalcolithic site of Dava Göz in NW IRAN using portable XRF. *Journal of Archaeological Science: Reports* **20**, 756–767 (2018).
103. Meyers, E. M., Meyers, C. L. & Gordon, B. D. *Sepphoris III: The Architecture, Stratigraphy, and Artifacts of the Western Summit of Sepphoris.* (Eisenbrauns, University Park, PA, 2018).
104. Weiss, Z. Sepphoris. in *Oxford Research Encyclopedia of Classics* (2017). doi:10.1093/acrefore/9780199381135.013.8039.
105. Bagnasco Gianni, G., Garzulino, A., Kay, S., Marzullo, M. & Smith, C. J. Civita di tarquinia (comune di tarquinia, provincia di viterbo, regione lazio). (2018) doi:10.1017/S0068246218000132.
106. Logvin, A. V. & Shevnina, I. V. Study of the Sintashta burial ground Karatomar, barrow 1 (preliminary report). in *Proceedings of the All-Russian Scientific Conference with International Participation* 123–125 (Samara, 2018).
107. Sava, E. & Kaiser, E. Die Siedlung mit "Aschehügeln" beim Dorf Odaia-Miciurin, Republik Moldova. Archäologische und naturwissenschaftliche Untersuchungen. (2011).
108. Giscard, P.-H., Tsagaan, T. & Crubezy, E. *Le premier empire des steppes en Mongolie: histoire du peuple Xiongnu et étude pluridisciplinaire de l'ensemble funéraire d'Egyin Gol.* (Editions Faton, 2013).
109. Keyser-Tracqui, C., Crubézy, E. & Ludes, B. Nuclear and Mitochondrial DNA Analysis of a 2,000-Year-Old Necropolis in the Egyin Gol Valley of Mongolia. *Am J Hum Genet* **73**, 247–260 (2003).
110. Keyser, C. *et al.* Genetic evidence suggests a sense of family, parity and conquest in the Xiongnu Iron Age nomads of Mongolia. *Hum Genet* **140**, 349–359 (2021).
111. Lepetz, S., Bernard, V., Tȯrbat, T. & Cervel, M. *Occupations et espaces sacrés dans l'Altaï mongol: les sites archéologiques de Burgast et Ikh Khatuu (1100 a.C.-1100 p.C.) : résultats des fouilles (2014-2016) de la mission archéologique franco-mongole dirigée par Sébastien Lepetz et Tsagaan Turbat.* (Éditions Mergoil, 2022).
112. Iderkhangai, T.-O. *et al.* Zoolongiyn am site. in *Joint Mongolian-Russian Central Asian Archaeological Project. Report on archaeological excavations in 2017* 7–43 (The Department of Archaeology, Ulaanbaatar State University, Ulaanbaatar, 2017).
113. Szeliga, M. *et al.* Early farming settlement of the marginal zone of loess uplands and its palaeoenvironmental context – a case study of the Iłża Piedmont (S Poland). *Praehistorische Zeitschrift* **98**, 450–483 (2023).
114. Kuzmina O.V. & Lastovsky A.A. Parking lot Krasny Gorodok // Ancient cultures of the forest-steppe Volga region. (1995).
115. Čugunov, K. V., Parzinger, H. & Nagler, A. Der skythenzeitliche Fürstenkurgan Aržan 2 in Tuva. Archäologie in Eurasien 26. *Mainz, Germany: Philipp von Zabern (in German).[Google Scholar]* (2010).
116. Zaitseva, G. I. *et al.* Chronology and possible links between climatic and cultural change during the first millennium BC in southern Siberia and Central Asia. *Radiocarbon* **46**, 259–276 (2004).

117. Chugunov, K., Parzinger, H. & Nagler, A. Chronology and cultural affinity of the kurgan Arzhan-2 complex according to archaeological data. *Impact of the environment on human migration in Eurasia* 1–9 (2004).
118. Chugunov, K. V., Nagler, A. & Parzinger, H. *Discovery of a Royal Burial of the Scythian Period in Tuva.* (The Metropolitan Museum of Art, 2006).
119. Zvénigorosky, V. *et al.* The genetic legacy of legendary and historical Siberian chieftains. *Commun Biol* **3**, 1–9 (2020).
120. Librado, P. *et al.* Tracking the origins of Yakutian horses and the genetic basis for their fast adaptation to subarctic environments. *PNAS* **112**, E6889–E6897 (2015).
121. LT, Y. Kurgan-svyatilishche mogilnika Filippovka 2, rol i mesto zhivotnykh v pogrebalnom obryade. *Arkheologiya vostochnoevropeiskikh stepei* 305–311 (2013).
122. Belinskiy, A. B. & Dudarev, S. *L., 2007. K Datirovke Kompleksov Pozdneyshego Predskifskogo Vremeni Severnogo Kavkaza i Vostochnoy Evropy [On Dating of Assemblages of Latest Pre-Scythian Time in North Caucasus and Eastern Europe]. Materialy Po Izucheniyu Istoriko-Kul'turnogo Nslediya Severnogo Kavkaza [Materials on the Study of the Historical and Cultural Heritage of the North Caucasus]*. vol. VII (Moscow: Pamyatniki istoricheskov mysli, 2007).
123. Reinhold, S. *et al.* The Late Bronze Age in the North Caucasus high mountain zone and beyond. *Der Kaukasus zwischen Osteuropa und Vorderem Orient: Dialog der Kulturen, Kultur des Dialoges (zum 140 Gedenken an Alexander A Miller). Archäologie in Iran und Turan. Berlin: Dietrich Reimer Verlag* 405–55 (2020).
124. Belinskij, A., Kalmykov, A., Korenevskij, S. & Härke, H. The Ipatovo kurgan on the North Caucasus steppe (Russia). *Antiquity* **74**, 773–774 (2000).
125. Gavrilova, A. A. Excavations of the second Katandinsky burial ground. in *Soviet archeology* 250–268 (Academy of Sciences of the USSR, Moscow, 1957).
126. Plasteeva, N. A., Tishkin, A. A. & Sablin, M. V. Horses from the Large Katanda mound (Altai). in *Modern solutions of actual problems of the Eurasian archeology. Collection of scientific articles. Issue 2* (ed. Tishkin, A. A.) 107–109 (Altai State University, Barnaul, 2018).
127. Dashkovskiy, P. K. The burial ground of Pazyryk culture the Hankarinsky dol in Altai: characteristic of a funeral ceremony and main directions of interdisciplinary research. *Peoples and religions of Eurasia* 42–66 (2016).
128. Vasilyeva I.N. Report on the archaeological excavations of the burial mound Sadgorod IV in Kinel-Cherkassky district of the Samara region on the open sheet number. (2017).
129. Kuznetsov, P. The time of cultures of the Late Bronze Age of the Volga region (analysis of radiocarbon dating). in *Proceedings of the Fourth All-Russian Archaeological Congress* 230–236 (Kazan, 2014).
130. Tishkin, A. A. & Gorbunov, V. V. Study of funeral and memorial sites of nomads in the Central Altai. in *Problems of Archaeology Ethnography Anthropology of Siberia and Neighboring Territories* (eds. Derevianko, A. P. & Molodin, V. I.) vol. IX 488–493 (Institute of Archeology and Ethnography SB RAS, Novosibirsk, 2003).
131. Valenzuela-Lamas, S. *Alimentació i ramaderia al Penedès durant la protohistòria: segles VII-III aC.* (Societat Catalana d'Arqueologia, 2008).
132. García, Cristina. Informe de l'intervenció arqueològica preventiva al jaciment iberoromà de l'Hereuet (Seró, Artesa de Segre, La Noguera). (2007).
133. Martín, A., Casas, S., Falgàs, F. C. i, Sastre, J. M. i & Prado, G. de. La zona 14 de l'oppidum del puig de Sant Andreu d'Ullastret. Un conjunt arquitectònic dels segles IV i III AC. *Cypsela: revista de prehistòria i protohistòria* 265–284 (2005).
134. Näsman, U. & Wegraeus, E. *Eketorp. Fortification and Settlement on Öland/Sweden. The Setting.* (Royal Academy of Letters History and Antiquities, 1979).
135. Monikander, A. Våtmarkskult vid Skedemosse under järnåldern. (Stockholms universitet, 2009).
136. Дубова, Н. А. *et al.* SEVERAL RESULTS OF BIOARCHAEOLOGICAL AND ETHNOECOLOGICAL INVESTIGATIONS IN TAJIKISTAN. *1* **34**, 149–164 (2022).
137. Filimonova, T. G. Baljuan archaeological expedition. Excavations and exploration 2013–2014. in *Archaeological work in Tajikistan* 252–266 (2019).
138. Tishkin, A. A., Filimonova, T. G., Gorbunov, V. V. & Seregin, N. N. Shokhidon – collapsing necropolis of the Early Middle Ages in Tajikistan. *Preservation and study of the cultural heritage of Altai Krai* 304–311 (2019).

139. Makarowicz, P., Ilchyshyn, V., Pasicka, E. & Makowiecki, D. An Elite Bronze Age Double-Horse Burial from Western Ukraine and the Chariot Package Dissemination. *Journal of Field Archaeology* **48**, 19–35 (2023).
140. Fitzhugh, W. W. & Bayarsaikhan, J. American-Mongolian Deer Stone Project: Field Report 2009. (2010).
141. Fitzhugh, W. W., Kortuym, R. & Bayarsaikhan, J. Rock Art and Archaeology: Investigating Ritual Landscape in the Mongolian Altai 2012. (2013).
142. Taylor, W. T. T. *et al.* Origins of equine dentistry. *Proceedings of the National Academy of Sciences* **115**, E6707–E6715 (2018).
143. Taylor, W. T. T. *et al.* A Bayesian chronology for early domestic horse use in the Eastern Steppe. *Journal of Archaeological Science* **81**, 49–58 (2017).
144. Zazzo, A., Lepetz, S., Magail, J. & Gantulga, J.-O. High-precision dating of ceremonial activity around a large ritual complex in Late Bronze Age Mongolia. *Antiquity* **93**, 80–98 (2019).
145. Taylor, W. T. T. *et al.* Early dispersal of domestic horses into the Great Plains and northern Rockies. *Science* **379**, 1316–1323 (2023).
146. Jagannathan, V. *et al.* Comprehensive characterization of horse genome variation by whole-genome sequencing of 88 horses. *Animal Genetics* **50**, 74–77 (2019).
147. Der Sarkissian, C. *et al.* Evolutionary Genomics and Conservation of the Endangered Przewalski's Horse. *Current Biology* **25**, 2577–2583 (2015).
148. Schubert, M. *et al.* Prehistoric genomes reveal the genetic foundation and cost of horse domestication. *PNAS* **111**, E5661–E5669 (2014).
149. Librado, P. *et al.* Ancient genomic changes associated with domestication of the horse. *Science* **356**, 442–445 (2017).
150. Do, K.-T. *et al.* Genomic characterization of the Przewalski′s horse inhabiting Mongolian steppe by whole genome re-sequencing. *Livestock Science* **167**, 86–91 (2014).
151. Andersson, L. S. *et al.* Mutations in DMRT3 affect locomotion in horses and spinal circuit function in mice. *Nature* **488**, 642–646 (2012).
152. Cosgrove, E. J. *et al.* Genome Diversity and the Origin of the Arabian Horse. *Sci Rep* **10**, 9702 (2020).
153. Jónsson, H. *et al.* Speciation with gene flow in equids despite extensive chromosomal plasticity. *PNAS* **111**, 18655–18660 (2014).
154. Liu, X. *et al.* EPAS1 gain-of-function mutation contributes to high-altitude adaptation in Tibetan horses. *Mol Biol Evol* msz158 (2019) doi:10.1093/molbev/msz158.
155. Lee, W. *et al.* Analysis of cross-population differentiation between Thoroughbred and Jeju horses. *Asian-Australas J Anim Sci* **31**, 1110–1118 (2018).
156. Metzger, J. *et al.* Runs of homozygosity reveal signatures of positive selection for reproduction traits in breed and non-breed horses. *BMC Genomics* **16**, 764 (2015).
157. Wallner, B. *et al.* Y Chromosome Uncovers the Recent Oriental Origin of Modern Stallions. *Current Biology* **27**, 2029-2035.e5 (2017).
158. Schrimpf, R. *et al.* Screening of whole genome sequences identified high-impact variants for stallion fertility. *BMC Genomics* **17**, 288 (2016).
159. Park, W. *et al.* Investigation of de novo unique differentially expressed genes related to evolution in exercise response during domestication in Thoroughbred race horses. *PLoS One* **9**, e91418 (2014).
160. Jun, J. *et al.* Whole genome sequence and analysis of the Marwari horse breed and its genetic origin. *BMC Genomics* **15 Suppl 9**, S4 (2014).
161. Orlando, L. *et al.* Ancient DNA analysis. *Nat Rev Methods Primers* **1**, 1–26 (2021).
162. Danecek, P. *et al.* Twelve years of SAMtools and BCFtools. *GigaScience* **10**, giab008 (2021).
163. Molloy, E. K., Durvasula, A. & Sankararaman, S. Advancing admixture graph estimation via maximum likelihood network orientation. *Bioinformatics* **37**, i142–i150 (2021).
164. Warmuth, V. *et al.* European Domestic Horses Originated in Two Holocene Refugia. *PLOS ONE* **6**, e18194 (2011).
165. Gronau, I., Hubisz, M. J., Gulko, B., Danko, C. G. & Siepel, A. Bayesian inference of ancient human demography from individual genome sequences. *Nat Genet* **43**, 1031–1034 (2011).

166. Renaud, G. *et al.* Improved de novo genomic assembly for the domestic donkey. *Science Advances* **4**, eaaq0392 (2018).
167. Wade, C. M. *et al.* Genome sequence, comparative analysis, and population genetics of the domestic horse. *Science* **326**, 865–867 (2009).
