# Supplementary material for: Widespread horse-based mobility arose around 2200 bce in Eurasia
Source: Nature. 2024 Jun 6;631(8022):819–25. doi: 10.1038/s41586-024-07597-5 (PMC11269178; doi:10.1038/s41586-024-07597-5)
Supplement: Supplementary file 2 — Reporting Summary [file 41586_2024_7597_MOESM2_ESM.pdf]

nature portfolio

Corresponding author(s): Pablo Librado and Ludovic Orlando

Last updated by author(s): 22/3/24

Reporting Summary

Nature Portfolio wishes to improve the reproducibility of the work that we publish. This form provides structure for consistency and transparency in reporting. For further information on Nature Portfolio policies, see our Editorial Policies and the Editorial Policy Checklist.

Statistics

For all statistical analyses, confirm that the following items are present in the figure legend, table legend, main text, or Methods section.

n/a	Confirmed	
☐	☒	The exact sample size (n) for each experimental group/condition, given as a discrete number and unit of measurement
☒	☐	A statement on whether measurements were taken from distinct samples or whether the same sample was measured repeatedly
☐	☒	The statistical test(s) used AND whether they are one- or two-sided<br>*Only common tests should be described solely by name; describe more complex techniques in the Methods section.*
☐	☒	A description of all covariates tested
☐	☒	A description of any assumptions or corrections, such as tests of normality and adjustment for multiple comparisons
☐	☒	A full description of the statistical parameters including central tendency (e.g. means) or other basic estimates (e.g. regression coefficient) AND variation (e.g. standard deviation) or associated estimates of uncertainty (e.g. confidence intervals)
☐	☒	For null hypothesis testing, the test statistic (e.g. F, t, r) with confidence intervals, effect sizes, degrees of freedom and P value noted<br>*Give P values as exact values whenever suitable.*
☐	☒	For Bayesian analysis, information on the choice of priors and Markov chain Monte Carlo settings
☒	☐	For hierarchical and complex designs, identification of the appropriate level for tests and full reporting of outcomes
☐	☒	Estimates of effect sizes (e.g. Cohen's d, Pearson's r), indicating how they were calculated

Our web collection on statistics for biologists contains articles on many of the points above.

Software and code

Policy information about availability of computer code

Data collection	*Provide a description of all commercial, open source and custom code used to collect the data in this study, specifying the version used OR state that no software was used.*
Data analysis	QGIS 3.36 (https://www.qgis.org/en/site/about/index.html)<br>Angsd v0.927 (https://www.popgen.dk/angsd/index.php/ANGSD)<br>Plink v1.9 (https://www.cog-genomics.org/plink/1.9/)<br>Locator (https://github.com/kr-colab/locator)<br>Admixtools2 (https://uqrmaie1.github.io/admixtools/articles/admixtools.html)<br>Admixtools v7.0.2 (https://github.com/DReichLab/AdmixTools)<br>AdmixtureBayes (https://github.com/avaughn271/AdmixtureBayes)<br>Struct-f4 (https://bitbucket.org/plibradosanz/structf4/src/master/)<br>generation_time (https://bitbucket.org/plibradosanz/generationtime/src/master/)<br>GONE (https://github.com/esrud/GONE/)

For manuscripts utilizing custom algorithms or software that are central to the research but not yet described in published literature, software must be made available to editors and reviewers. We strongly encourage code deposition in a community repository (e.g. GitHub). See the Nature Portfolio guidelines for submitting code & software for further information.

Data

Policy information about availability of data

All manuscripts must include a data availability statement. This statement should provide the following information, where applicable:

- Accession codes, unique identifiers, or web links for publicly available datasets
- A description of any restrictions on data availability
- For clinical datasets or third party data, please ensure that the statement adheres to our policy

All collapsed and paired-end sequence data for samples sequenced in this study are available in compressed FASTQ format through the European Nucleotide Archive under accession number PRJEB71445, together with rescaled and trimmed bam sequence alignments against both the nuclear horse reference genomes. Previously published ancient data used in this study are available under accession numbers PRJEB7537, PRJEB10098, PRJEB10854, PRJEB22390, PRJEB31613, and PRJEB44430, and detailed in Supplementary Table 1. The genomes of 78 modern horses, publicly available, were also accessed as indicated in their corresponding original publications, and in Supplementary Table 1.

Research involving human participants, their data, or biological material

Policy information about studies with human participants or human data. See also policy information about sex, gender (identity/presentation), and sexual orientation and race, ethnicity and racism.

Reporting on sex and gender	*Use the terms sex (biological attribute) and gender (shaped by social and cultural circumstances) carefully in order to avoid confusing both terms. Indicate if findings apply to only one sex or gender; describe whether sex and gender were considered in study design; whether sex and/or gender was determined based on self-reporting or assigned and methods used. Provide in the source data disaggregated sex and gender data, where this information has been collected, and if consent has been obtained for sharing of individual-level data; provide overall numbers in this Reporting Summary. Please state if this information has not been collected. Report sex- and gender-based analyses where performed, justify reasons for lack of sex- and gender-based analysis.*
Reporting on race, ethnicity, or other socially relevant groupings	*Please specify the socially constructed or socially relevant categorization variable(s) used in your manuscript and explain why they were used. Please note that such variables should not be used as proxies for other socially constructed/relevant variables (for example, race or ethnicity should not be used as a proxy for socioeconomic status). Provide clear definitions of the relevant terms used, how they were provided (by the participants/respondents, the researchers, or third parties), and the method(s) used to classify people into the different categories (e.g. self-report, census or administrative data, social media data, etc.) Please provide details about how you controlled for confounding variables in your analyses.*
Population characteristics	*Describe the covariate-relevant population characteristics of the human research participants (e.g. age, genotypic information, past and current diagnosis and treatment categories). If you filled out the behavioural & social sciences study design questions and have nothing to add here, write "See above."*
Recruitment	*Describe how participants were recruited. Outline any potential self-selection bias or other biases that may be present and how these are likely to impact results.*
Ethics oversight	*Identify the organization(s) that approved the study protocol.*

Note that full information on the approval of the study protocol must also be provided in the manuscript.

Field-specific reporting

Please select the one below that is the best fit for your research. If you are not sure, read the appropriate sections before making your selection.

☐ Life sciences ☐ Behavioural & social sciences ☒ Ecological, evolutionary & environmental sciences

For a reference copy of the document with all sections, see nature.com/documents/nr-reporting-summary-flat.pdf

Ecological, evolutionary & environmental sciences study design

All studies must disclose on these points even when the disclosure is negative.

Study description	We generate 124 new ancient genomes, and combined them with 434 previously available to identify when domestic horses were extensively used for long-distance mobility
Research sample	The 550 horse genomes used in this study were used to provide a comprehensive representation of the horse genetic diversity prior and during domestication across Eurasia. The rationale was to identify shifts in the evolutionary trajectory of horses induced by humans, following their domestication. Eight outgroups were also included to polarise alleles as ancestral or derived. A full description of each new sample is provided in Table S1.
Sampling strategy	In the field of ancient DNA, sampling strategies are conditioned by the levels of endogenous DNA preservation in ancient remains. We attempted to (and succeeded) generate the largest genomic time-series for a non-human species. This was larger than our own

	previous studies (eg. Librado et al. 2021), where the corresponding sampling size was already proven more than sufficient to perform evolutionary analyses
Data collection	Fossil remains were collected from across Eurasia, from coauthors of this study. Sequencing of these remains was performed at the dedicated facilities of CAGT (Toulouse). The contribution of each coauthor is detailed in the corresponding section of the main manuscript, and the whole process was registered in our internal database.
Timing and spatial scale	Ancient DNA samples were processed for whole genome sequenced as they arrived to our laboratory, shipped by our coauthors. No particular strategy was followed in this regard.
Data exclusions	All data included in this study was analysed, with the only exception pertaining to a few samples showing signatures of introgression from basal lineages (true outliers) or not radiocarbon-dated in the regression analyses, as openly explained in the supplementary information.
Reproducibility	All our experiments were found to be highly reproducible. Those experiments with lower reproducibility, pertaining to complex statistical inference, were repeated multiple times with different starting values and parameters to check for concordance between runs (eg. Locator runs). Only solid analyses are reported.
Randomization	We followed the population group assignment from Librado et al. (2021; Nature)
Blinding	Blinding was not relevant in our ancient DNA study.

Did the study involve field work? ☒ Yes ☐ No

Field work, collection and transport

Field conditions	Fossil remains were collected across Eurasia during years, if not decades, during a diversity of conditions. These do not impact our conclusions as fossils have been buried for hundreds to thousands of years before being sampled during field work.
Location	All relevant parameters, including radiocarbon dates and GPS coordinates for each new ancient sample sequenced in this study, are provided in Table S1
Access & import/export	All fossils were collected strictly following the highest standards in ancient DNA research, and in close coordination with the archaeologists responsible for the material and the corresponding excavations, with all local and international permits in place. All these archaeologists are coauthors in our study.
Disturbance	This study caused no disturbance

Reporting for specific materials, systems and methods

We require information from authors about some types of materials, experimental systems and methods used in many studies. Here, indicate whether each material, system or method listed is relevant to your study. If you are not sure if a list item applies to your research, read the appropriate section before selecting a response.

Materials & experimental systems

n/a	Involved in the study
☒	☐ Antibodies
☒	☐ Eukaryotic cell lines
☐	☒ Palaeontology and archaeology
☒	☐ Animals and other organisms
☒	☐ Clinical data
☒	☐ Dual use research of concern
☒	☐ Plants

Methods

n/a	Involved in the study
☒	☐ ChIP-seq
☒	☐ Flow cytometry
☒	☐ MRI-based neuroimaging

Palaeontology and Archaeology

Specimen provenance	The samples that were analyzed in this study were collected from a range of archaeological contexts, as detailed in the Supplementary Information and summarized in Supplementary Table S1. As this involved sampling from across Eurasia and different procedures between countries and institutions, key contact persons were identified in each country so as to access relevant material and coordinate legal authorization to sample material for DNA analysis and radiocarbon dating. Samples were collected with permission from the organizations holding the collections and documented through official agreement letters provided by the named archaeologists and/or curators and/or directors of relevant institutions, named below. As DNA and radiocarbon dating techniques are partially destructive, we sought every opportunity to access samples as part of collaborations with other research projects so as to both save resources and avoid double sampling, and, thus, ultimately minimize destruction. The following list provides the sites and

names of those key contact persons, who granted access to the corresponding material, with reference to letters and permits where appropriate:

-Albufeira (Silo 1, Rua Henrique Calado), Portugal. Sample: Albufeira1x2_Spa_1224. Key contacts: Maria João Valente, and Luís Paulo (Museu Municipal de Arqueologia, Albufeira). Collection of the Museu Municipal de Arqueologia, Albufeira.

-Alorda Park, Calafell, Spain. Sample: H9020_Spa_m291 (SU 9020 - Bottom level of the filling of the ditch surrounding the fortified aristocratic residence). Key contact: Silvia Valenzuela-Lamas (Archaeology of Social Dynamics, Institució Milà i Fontanals - Consejo Superior de Investigaciones Científicas (IMF-CSIC), C/ Egipcíaques 15, 08001 Barcelona, Spain).

-Arzhan-2, Russia. Sample: Rus8_Rus_m855. Key contacts: Aleksei K. Kasparov, Vladimir V. Pitulko (Institute of Material Culture, Russian Academy of Sciences). Sampled through the project 21-18-00457 from the Russian Science Foundation, with permission and all proper authority (confirmation letter nb 14102/33-772.4-263).

-At Daban, Yakutia. Sample: ATDABAN13_Yak_1725. Key contact: Éric Crubézy (Centre d'Anthropobiologie et de Génomique de Toulouse, CNRS UMR 5288, Université Paul Sabatier, Faculté de Médecine Purpan, 37 Allées Jules Guesde, 31000 Toulouse, France).

-Bakonszeg-Kádárdomb, Hungary. Sample: BAK1_Hun_m1686. Key contacts: Lajos Lakner, Dani János, Katherine Kanne (Department of Archaeology and History, University of Exeter, Exeter EX4 4QE, UK). Exported in 2008-2009 under National Science Foundation Dissertation Improvement Grant nb 0833106, Wenner-Gren Foundation for Anthropological Research Dissertation Fieldwork Grant nb 7896). Collections Institution: Déri Museum.

-Berettyóújfalu-Szilhalom, Hungary. Sample: BS11_Hun_m1682. Key contacts: Lajos Lakner, Dani János, Katherine Kanne (Department of Archaeology and History, University of Exeter, Exeter EX4 4QE, UK). Exported in 2008-2009 under National Science Foundation Dissertation Improvement Grant nb 0833106, Wenner-Gren Foundation for Anthropological Research Dissertation Fieldwork Grant nb 7896). Collections Institution: Déri Museum.

-Biluut 2, Zeerdegchingiin Khoshuu, Zunii Gol, Zuunkhangai, Ulaan Tolgoi, Mongolia: Samples M17x152x1_Mon_m80 and M17x156_Mon_m299. Key contact: Jamsranjav Bayarsaikhan (Institute of Archaeology, Mongolian Academy of Science, Ulaanbaatar 13330, Mongolia), and Will T. T. Taylor (Museum of Natural History, University of Colorado Boulder, Boulder, CO 80309, USA; Fulbright US Student research award nb 34154234, National Geeographic Young Explorer's grant nb 9713-15), National Science Foundation Doctoral Dissertation Improvement Grant nb 1522024). Exported in 2015 and 2015 under research agreement nb 20150315. Collections from the National Museum of Mongolia, Ulaanbaatar

-Bitozeves, Czechia. Sample: PRA29_Cze_471. Key contact: René Kyselý, Institute of Archaeology of the Czech Academy of Sciences, Prague. Excavated by Věra Šušická, ÚAPPSZČ, Most, Czechia.

-Bleachfield Street, Alcester, Warwickshire, United Kingdom. Key contact: Jacobo Weinstock (Faculty of Arts and Humanities, Department of Archaeology, University of Southampton, UK). Collection from the site curated by Warwickshire Museum.

-Bled, Pristava necropolis, Slovenia. Samples: SRSLO012_Slo_m197. Key contact: Peter Turk (Narodni muzej Slovenije, Prešernova 20, SI-1000 Ljubljana, Slovenia). Samples were made available through the Innovation Fund of the Austrian Academy of Sciences (ÖAW) (Grant agreement IF_2015_17).

-Borly IV, Kazakhstan. Sample: BorlyXIII_Kaz_m4638 and Borly9_Kaz_1807 (SQ 7G (33-44cm)). Key contact: Alan Outram (Department of Archaeology and History, University of Exeter, Exeter EX4 4QE, UK). Collections from the A. Kh. Margulan Joint Archaeological Research Centre Toraighyrov University (Director: Viktor K. Merts), sampled with permission and all proper authority from the Acting Deputy Chairman of the Board for Academic Work (confirmation letter 0605-2021 nb. 107-1232).

-Botai, Kazakhstan. Samples: Botai18x30_Kaz_m3328 and BotaiB_Kaz_m3228. Key contact: Prof Alan Outram (Department of Archaeology and History, University of Exeter, Exeter EX4 4QE, UK). Collections from the Al Farabi Kazakh National University, sampled with permission and all proper authority from the Dean of Faculty of History M.S. Nogaibayeva (confirmation letter 605-2021 nb. 1523-602).

-Bradgate Park, Leicestershire, United Kingdom. Sample: BGPSK1_UK_1661. Key contact: Richard Thomas (School of Archaeology and Ancient History, University of Leicester). Permission for analysis was provided by the Bradgate Park Trust. The specimen is curated at the School of Archaeology and Ancient History, University of Leicester, but will eventually be deposited with Leicestershire County Council Museums Service under the accession code XA19.2015.

-Bredholm, Denmark. Samples: P128_Den_1458, P129_Den_1457, P130_Den_1459, and P131_Den_1461. Key contact: Peter Pentz (museum inspector; National Museum of Denmark, Ny Vestergade 10, 1471 Copenhagen K., Denmark). Sampled with permission from the collection manager at the Collections of the Natural History Museum of Denmark.

-Brusyany IV, Kurgan 2, mound fill, Russia. Sample: LR18x70_Rus_608. Key contact: Pavel Kuznetsov (Department of Russian History and Archaeology, Samara State University of Social Sciences and Education, Samara, Russia). Sampled with proper authorization (confirmation letter nb 03-01-Myzea).

-Brusyany IV, Kurgan 1, Russia. Sample: LR18x68_Rus_592 (Grave 1, horse 1). Key contact: Pavel Kuznetsov (Department of Russian History and Archaeology, Samara State University of Social Sciences and Education, Samara, Russia). Sampled with proper authorization (confirmation letter nb 03-01-Myzea).

-Budapest-Királyok Útja 293, Hungary. Samples KU1153_Hun_m2301, KU1591_Hun_m2304, KU1701_Hun_m1822, KU2102_Hun_m2211, KU2210_Hun_m2218, and KU709_Hun_m2335. Key contacts: Paula Zsidi, Alice Choyke, Katherine Kanne (Department of Archaeology and History, University of Exeter, Exeter EX4 4QE, UK). Exported in 2008-2009 under National Science Foundation Dissertation Improvement Grant nb 0833106, Wenner-Gren Foundation for Anthropological Research Dissertation Fieldwork Grant nb 7896). Collections Institution: Aquincum Museum.

-Burgast, Mongolia: Samples GVA9046_Mon_716. Key contacts: Sébastien Lepetz (CNRS, Muséum national d'Histoire naturelle, Archéozoologie, Archéobotanique (AASPE), CP 56, Paris, France), Tsagaan Turbat (Archaeological Research Center and Department of Anthropology and Archaeology, National University of Mongolia, Ulaanbaatar, Mongolia), and Bayarkhuu Noost (Archaeological Research Center and Department of Anthropology and Archaeology, National University of Mongolia, Ulaanbaatar, Mongolia). Excavation campaign from 2016, Program MEAE – Institut of Archaeology, Mongolian Academy of Sciences, Ulaanbaatar, Mongolia.

-Çadır Höyük, Yozgat, Türkiye. Sample: CD5041_Tur_m314. Key contact: Benjamin Arbuckle (Department of Anthropology, Alumni Building, University of North Carolina at Chapel Hill, Chapel Hill, NC, USA). Exported in 2013 to Benjamin Arbuckle via the Yozgat Müze Müdürlüğü under grant (NSF BCS-1311551).

-Can Roqueta-Torre Romeu, Spain. Sample: CRTR279_Spa_506 (Barcelona, Spain - Late Roman, structure CRTR-279). Key contact: Silvia Albizuri (Institut d'Arqueologia, Universitat de Barcelona).

-Chekon settlement, Russia. Sample: KUZ3_Rus_m954. Key contact: Pavel Kuznetsov (Department of Russian History and Archaeology, Samara State University of Social Sciences and Education, Samara, Russia). Sampled with proper authorization (confirmation letter nb 03-01-Myzea).

-Dava Goz, Iran. Sampled: DavaG1_Ira_m4615. Key contact: Marjan Mashkour (CNRS, Muséum national d'Histoire naturelle, Archéozoologie, Archéobotanique (AASPE), CP 56, Paris, France).

-Derkul, Russia. Sample: NB4_Kaz_m4210. Key contacts: Pavel Kosintsev (Institute of Plant and Animal Ecology, Ural Branch of the Russian Academy of Sciences, Ekaterinburg, Russia), and Mélanie Pruvost (UMR5199, PACEA, Université de Bordeaux, France).

-Dunakeszi-Székesdűlő, Hungary. Samples: DS101_Hun_m2271 and DS113_Hun_m2222. Key contacts: Paula Zsidi, Alice Choyke, Katherine Kanne (Department of Archaeology and History, University of Exeter, Exeter EX4 4QE, UK). Exported in 2008-2009 under National Science Foundation Dissertation Improvement Grant nb 0833106, Wenner-Gren Foundation for Anthropological Research Dissertation Fieldwork Grant nb 7896). Collections Institution: Aquincum Museum.

-Eketorp, Sweden. Sample: F612_Swe_495. Key contacts: Key contacts: Johnny Karlsson (curator, The Swedish History Museum), Marie Sundquist (Östra Greda Research Group, 38791 Borgholm, Sweden), and Gabriella Lindgren (Department of Animal Breeding and Genetics, Swedish University of Agricultural Sciences, Uppsala, Sweden). Loan agreement: 331-2017-958.

-Egying Gol, Mongolia. Samples: EGI10_Mon_17 (Tomb 10), EGI12x2_Mon_41 (Tomb 12.2), EGI67_Mon_14 (Tomb 67), and EGI69_Mon_75 (Tomb 69). Key contacts: Éric Crubézy (Centre d'Anthropobiologie et de Génomique de Toulouse, CNRS UMR 5288, Université Paul Sabatier, Faculté de Médecine Purpan, 37 Allées Jules Guesde, 31000 Toulouse, France), and Tsagaan Turbat (Archaeological Research Center and Department of Anthropology and Archaeology, National University of Mongolia, Ulaanbaatar, Mongolia).

-El Graell (Vic), Spain. Sample: ADNUB33_Spa_m64 (Ibero Roman, Spain; structure E-42). Key contacts: F. Javier López-Cachero (Can Roqueta Project Manager, Ref: ARQ001SOL-178-2022), and Silvia Albizuri (Institut d'Arqueologia, Universitat de Barcelona).

-Filippovka II, Kurgan 1, Grave 2, Russia. Sample: LR18x84_Rus_m294. Key contact: Pavel Kuznetsov (Department of Russian History and Archaeology, Samara State University of Social Sciences and Education, Samara, Russia). Sampled with proper authorization (confirmation letter nb 03-01-Myzea).

-Følenslev Mose, Denmark. Sample: P133_Den_O1663. Key contact: Peter Pentz (museum inspector; National Museum of Denmark, Ny Vestergade 10, 1471 Copenhagen K, Denmark). Sampled with permission from the collection manager at the Collections of the Natural History Museum of Denmark.

-Gáborján-Csapszékpart, Hungary. Samples: GC77486_Hun_m1681 and GC77550_Hun_m1751. Key contacts: Lajos Lakner, Dani János, Katherine Kanne (Department of Archaeology and History, University of Exeter, Exeter EX4 4QE, UK). Exported in 2008-2009 under National Science Foundation Dissertation Improvement Grant nb 0833106, Wenner-Gren Foundation for Anthropological Research Dissertation Fieldwork Grant nb 7896). Collections Institution: Déri Museum.

-Ginnerup, Denmark. Samples: GIN1020_Den_m3000 (structure A4), GIN1055_Den_m3000 (structure A4), GIN396_Den_m3000 (structure A1), GIN489_Den_m3000 (structure A1), GIN561_Den_m3000 (structure A1). Key contact: Lutz Klassen (Museum Østjylland, Randers, Denmark).

-Gørlev, Denmark. Samples P187_Den_1334. Key contact: Peter Pentz (museum inspector; National Museum of Denmark, Ny Vestergade 10, 1471 Copenhagen K, Denmark). Sampled with permission from the collection manager at the Collections of the Natural History Museum of Denmark.

-Halvay, Kazakhstan. Sample: Halvai4_Kaz_342. Key contact: Prof Alan Outram (Department of Archaeology and History, University of Exeter, Exeter EX4 4QE, UK). Sampled with permission and all proper authority from the Acting Vice-Rector on Science, Internationalization and Digitalization Gulshat Shaikamal (confirmation letter 11.05.2021 nb. 15-20-09/1052). Collections from the A. Baitursynov Kostanay State University, KSU (Kostanay).

-Hereuet, Seró, Spain. Sample: H2012x137_Spa_m204 (SU 2012 - Filling of silo SJ-8). Key contact: Silvia Valenzuela-Lamas (Archaeology of Social Dynamics, Institució Milà i Fontanals - Consejo Superior de Investigaciones Científicas (IMF-CSIC), C/ Egipcíaques 15, 08001 Barcelona, Spain).

-Hjortspringkobbel, Denmark. Sample: P137_Den_m291. Key contact: Collections of the Natural History Museum of Denmark). Key contact: Kristian M. Gregersen (former collection manager of Quaternary Zoology, Natural History Museum of Denmark, Gothersgade 130, 1123 Copenhagen K., Denmark). Sampled with permission from the collection manager at the Collections of the Natural History Museum of Denmark.

-Hovmarken, Denmark. Sample: P138_Den_1224. Key contact: Kristian M. Gregersen (former collection manager of Quaternary Zoology, Natural History Museum of Denmark, Gothersgade 130, 1123 Copenhagen K., Denmark). Sampled with permission from the collection manager at the Collections of the Natural History Museum of Denmark.

-Hungate, United Kingdom. Sample: VEM107_UK_956. Key contact: Terry O'Connor (Department of Archaeology, University of York, c/o Kings Manor, York YO1 7EP, UK).Excavated by York Archaeological Trust between 2006 and 2011 from whom permission was granted to Terry O'Connor, University of York, for ancient DNA study in 2013 under exit documentation X0903 and X0937.

-Husiatyn, Ukraine. Sample: POZ54_Ukr_m1498 (Double burial). Key Contact: Daniel Makowiecki (Institute of Archaeology, Faculty of History, Nicolaus Copernicus University, Toruń, Poland). Two horse skeletons were discovered during rescue excavations (2015) of barrow by Vasyl Ilchyshyn (Zaliztsi Museum, Ternopil Region, Ukraine, and the Security Archaeological Service of the Institute of Archeology, National Academy of Sciences of Ukraine, Kyiv, Ukraine). Samples were collected during zooarchaeological research (2021) by Daniel Makowiecki and Przemysław Makrowicz (Faculty of Archaeology Adam Mickiewicz University, Poznań), and stored since at the Institute of Archaeology, Nicolaus Copernicus, Torun. One of the two horses was sampled for the DNA and radiocarbon analysis presented in this study.

-Idzhil, Russia. Sample: IDZH_Rus_734. Marjan Mashkour (CNRS, Muséum national d'Histoire naturelle, Archéozoologie, Archéobotanique (AASPE), CP 56, Paris, France).

-Industriya, Russia. Sample: KAU27B_Rus_m627. Key contacts: Sabine Reinhold and Svend Hansen (Eurasia Department of the German Archaeological Institute, Berlin, Germany). Excavation carried out by Dr. D. S. Korobov (Institue of Archaeology, Russian Academy of Sciences, Moscow, Russia, licence nb 2001-868), with material curated by Ltd. 'Nasledie'. Exported in 2016 with proper authorization to the German Archaeological Institute, Berlin, Germany.

-Ipatovo 3, Russia. Sample: KAU22_Rus_m877 (Kurgan 2, Animal Complex 13). Key contacts: Sabine Reinhold and Svend Hansen (Eurasia Department of the German Archaeological Institute, Berlin, Germany). Excavation carried out by Dr. A.B. Belinskij (Stavropol, excavation Ltd. 'Nasledie' & DAI, Eurasia-Department, license nb 1998-177). Exported in 2013 with proper authorization to the German Archaeological Institute, Berlin, Germany.

-Katanda II, Russia. Samples: Kat2x4_Rus_m112 and Katx11_Rus_m269. Key contact: Alexey A. Tishkin (Department of Archaeology, Ethnography and Museology, Altai State University, Prospekt Lenina, 61, 656049 Barnaul, Russia). Sampled with proper permission and authority by Alexey A. Tishkin from auxiliary collection of the Department of Archaeology, Ethnography and Museology of the Altai State University, under the framework of the Russian Science Foundation project "The world of ancient nomads of Inner Asia: interdisciplinary studies of material culture, sculptures and economy" (No. 22-18-00470).

-Karatomar Burial ground, Kazakhstan (Kurgan 1). Sample: Karat17039_Kaz_m1834. Key contact:
Prof Alan Outram (Department of Archaeology and History, University of Exeter, Exeter EX4 4QE, UK). Sampled with permission and all proper authority from the Acting Vice-Rector on Science, Internationalization and Digitalization Gulshat Shaikamal (confirmation letter 11.05.2021 nb. 15-20-09/1052). Collections from the A. Baitursynov Kostanay State University, KSU (Kostanay).

-Khankarinsky dol, Russia. Sample: Han12_Rus_m296. Key contact: Alexey A. Tishkin (Department of Archaeology, Ethnography and Museology, Altai State University, Prospekt Lenina, 61, 656049 Barnaul, Russia). Sampled with proper permission and authority by Alexey A. Tishkin from auxiliary collection of the Department of Archaeology, Ethnography and Museology of the Altai State University, under the framework of the Russian Science Foundation project "The world of ancient nomads of Inner Asia: interdisciplinary studies of material culture, sculptures and economy" (No. 22-18-00470).

-Kittsee settlement, Steinfeldäcker, Austria. Sample: KT46_Aus_m3240 (campaign 1997, pit 289). Key contact: Christian Mayer (Federal Monuments Authority Austria, Department for Digitalization and Knowledge Transfer, Vienna, Austria). Excavation documentation and excavated material accessible through the Federal Monuments Authority Austria, Department of Archaeology.

-Køge A ved Spanager, Denmark. Sample: P134_Den_862. Key contact: Kristian M. Gregersen (former collection manager of Quaternary Zoology, Natural History Museum of Denmark, Gothersgade 130, 1123 Copenhagen K., Denmark). Sampled with permission from the collection manager at the Collections of the Natural History Museum of Denmark.

-Krasnosamarskoe settlement, Russia. Sample: RN85_Rus_865. Key contact: Pavel Kuznetsov (Department of Russian History and Archaeology, Samara State University of Social Sciences and Education, Samara, Russia). Sampled with proper authorization (confirmation letter nb 03-01-Myzea).

-Krasnosamarskoe IV, kurgan cemetery, Kurgan 7, Russia. Sample: RN10_Rus_1131 (horse bone in the mound fill layer). Key contact: Pavel Kuznetsov (Department of Russian History and Archaeology, Samara State University of Social Sciences and Education, Samara, Russia). Sampled with proper authorization (confirmation letter nb 03-01-Myzea).

-Krasny Gorodok settlement, Russia. Sample: ABA3_Rus_1333. Key contact: Pavel Kuznetsov (Department of Russian History and Archaeology, Samara State University of Social Sciences and Education, Samara, Russia). Sampled with proper authorization (confirmation letter nb 03-01-Myzea).

-Krefeld-Gellep, Germany. Sample Kref4_Ger_48. Key contact: Sabine Deschler-Erb and Monika Schernig Mráz (Integrative Prehistory and Archaeological Science, University Basel). Sample made available through Project HumAnimAl: Swiss National Science Foundation 178834. Sample reference NI 2017/0030 2708-12, Museum Burg Linn, Germany.

-Le Cendre – Gondole, France: Samples GVA629_Fra_m112, and GVA636_From_m197. Key contact: Sébastien Lepetz (Muséum national d'Histoire naturelle, Archéozoologie, Archéobotanique (AASPE), CP 56, Paris, France). Excavation campaign from 2003.

-Langhøj, Denmark. Samples P189_Den_1541. Key contact: Kristian M. Gregersen (former collection manager of Quaternary Zoology, Natural History Museum of Denmark, Gothersgade 130, 1123 Copenhagen K., Denmark). Sampled with permission from the

collection manager at the Collections of the Natural History Museum of Denmark.

-Maison Alfort Museum of the Veterinarian School, France (7 Av du Général De Gaulle, 94704 Maisons Alfort, France). Sample: Alfort3_Fra_1806: Key contacts: Christophe Degueurce, and Céline Robert (Ecole Nationale Vétérinaire d'Alfort, 7 Avenue du Général De Gaulle, 94704 Maisons-Alfort, France). Sampled with permission from the collections of the Maison Alfort Museum of the Veterinarian School.

-Miciurin (Odaia), Moldavia. Sample: Miciurin01_Mol_794. Key contact: Arne Ludwig (Leibniz-Zentrum für Archäologie (LEIZA), Ludwig-Lindenschmit-Forum 1, 55116 Mainz, Germany).

-Noviye Kluchi III cemetery, Bronze Age Pokrovka Culture, Kurgan 1, sacrificial complex, Russia. Sample: LR18x3_Rus_m1820. Key contact: Pavel Kuznetsov (Department of Russian History and Archaeology, Samara State University of Social Sciences and Education, Samara, Russia). Sampled with proper authorization (confirmation letter nb 03-01-Myzea).

-Nytorv, Denmark. Sample: P139_Den_1381. Key contact: Kristian M. Gregersen (former collection manager of Quaternary Zoology, Natural History Museum of Denmark, Gothersgade 130, 1123 Copenhagen K., Denmark). Sampled with permission from the collection manager at the Collections of the Natural History Museum of Denmark.

-Orcet – La Roche Blanche – L'Enfer, France: Samples GVA637_Fra_m64 and GVA639_Fra_2. Key contact: Sébastien Lepetz (CNRS, Muséum national d'Histoire naturelle, Archéozoologie, Archéobotanique (AASPE), CP 56, Paris, France). Excavation campaign from 2003.

-Pech Maho, France. Sample: Pech126_Fra_m288. Key contact: Armelle Gardeisen (CNRS, Archéologie des Sociétés Méditerranéennes, Archimède IA-ANR-11-LABX-0032-01, Université Paul Valéry, Montpellier 34090, France).

-Puig de Sant Andreu, Spain (Ullastret). Sample: UE14029_Spa_m293 (SU14029 - abandoned layer of the main residence; zona 14 of this urban site of more than 10Ha). Key contact: Silvia Valenzuela-Lamas (Archaeology of Social Dynamics, Institució Milà i Fontanals - Consejo Superior de Investigaciones Científicas (IMF-CSIC), C/ Egipcíaques 15, 08001 Barcelona, Spain).

-Rathewitz 13, Burgenland district, Saxony-Anhalt, Central Germany. Sample: Rat13_Ger_474 (deposited in grave 13 of a burial ground from the Migration period, 5th/6th century). Key contact: Hans-Jürgen Döhle (former curator of State Museum of Prehistory, Halle (Saale), Germany). Stored in the State Office for Heritage Management and Archaeology Saxony-Anhalt - State Museum of Prehistory, Halle (Saale), Germany.

-Rislev, Denmark. Sample: P188_Den_149. Key contact: Kristian M. Gregersen (former collection manager of Quaternary Zoology, Natural History Museum of Denmark, Gothersgade 130, 1123 Copenhagen K., Denmark). Sampled with permission from the collection manager at the Collections of the Natural History Museum of Denmark.

-Roseldorf, Austria. Samples: SRNHM007_Aus_m301 and SRNHM004_Aus_m294. Key contact: Erich Pucher (Naturhistorisches Museum Wien, Austria). Samples were made available through the Innovation Fund of the Austrian Academy of Sciences (ÖAW) (Grant agreement IF_2015_17).

-Sadgorod IV, Kurgan 2, sacrificial complex 2, Russia. Sample: LR18x5_Rus_1810. Key contact: Pavel Kuznetsov(Department of Russian History and Archaeology, Samara State University of Social Sciences and Education, Samara, Russia). Sampled with proper authorization (confirmation letter nb 03-01-Myzea).

-Sarengrad-Klopare, Croatia. Samples: SRKRO001_Cro_720 and SRKRO002_Cro_722. Key contact: Mario Novak (Centre for Applied Bioanthropology, Institute for Anthropological Research, Ljudevita Gaja 32, 10 000 Zagreb, Croatia), and Andrea Rimpf. (Ilok Town Museum, Šetalište o. Mladena Barbarića 5, 32236 Ilok, Croatia). Samples were made available through the Innovation Fund of the Austrian Academy of Sciences (ÖAW) (Grant agreement IF_2015_17).

-Százhalombatta-Földvár, Hungary. Samples: SZHB2027_Hun_m2033, SZHB2074_Hun_m2115, SZHB2079_Hun_m1575, SZHB2147_Hun_m2054, SZHB2158_Hun_m1820, SZHB2438_Hun_m1602, SZHB553_Hun_m1566, SZHB625_Hun_m984, SZHB734_Hun_m1576, and SZHB967_Hun_m1822. Key Contact: Magdolna Vicze, Katherine Kanne (Department of Archaeology and History, University of Exeter, Exeter EX4 4QE, UK). Exported in 2008-2009 under National Science Foundation Dissertation Improvement Grant nb 0833106, Wenner-Gren Foundation for Anthropological Research Dissertation Fieldwork Grant nb 7896). Collections Institution: Matrica Museum.

-Sepphoris, Israel. Sample: MV243_Isr_294. Key contact: Liora Kolska Horwitz (National Natural History Collections, Edmond J. Safra Campus, Givat Ram, The Hebrew University; Jerusalem 9190401, Israel) Sampled with permission and all proper authority from Zeev Weiss (Eleazar L. Sukenik Professor of Archaeology, Institute of Archaeology, The Hebrew University of Jerusalem), archaeologist in charge of site.

-Shanmava, China. Sample: Shanx1_Chi_m112. Key contact: Alexey A. Tishkin (Department of Archaeology, Ethnography and Museology, Altai State University, Prospekt Lenina, 61, 656049 Barnaul, Russia). Sampled with proper permission and authority by Alexey A. Tishkin from auxiliary collection of the Department of Archaeology, Ethnography and Museology of the Altai State University, under the framework of the Russian Science Foundation project "The world of ancient nomads of Inner Asia: interdisciplinary studies of material culture, sculptures and economy" (No. 22-18-00470).

-Shahr-i-Qumis, Iran. Sample: AM3_Ira_Modern. Key contact: Marjan Mashkour (CNRS, Muséum national d'Histoire naturelle, Archéozoologie, Archéobotanique (AASPE), CP 56, Paris, France).

-Shohidon, Tajikistan. Sample: Shohx1_Rus_720 (Grave 20). Key contact: Alexey A. Tishkin (Department of Archaeology, Ethnography and Museology, Altai State University, Prospekt Lenina, 61, 656049 Barnaul, Russia). Sampled with proper permission and authority by Alexey A. Tishkin from auxiliary collection of the Department of Archaeology, Ethnography and Museology of the Altai State University, under the framework of the Russian Science Foundation project "The world of ancient nomads of Inner Asia:

interdisciplinary studies of material culture, sculptures and economy" (No. 22-18-00470).

-Shumaevo I, Kurgan 5, ditch, Russia. Samples: LR18x65_Rus_1352 (SE sector, skull 5) and LR18x66_Rus_1353 (NE sector, Skull 1, depth -123). Key contact: Pavel Kuznetsov (Department of Russian History and Archaeology, Samara State University of Social Sciences and Education, Samara, Russia). Sampled with proper authorization (confirmation letter nb 03-01-Myzea).

-Skedemosse, Sweden. Sample: F139_Swe_342. Key contacts: Johnny Karlsson (curator, The Swedish History Museum), Marie Sundquist (Östra Greda Research Group, 38791 Borgholm, Sweden), and Gabriella Lindgren (Department of Animal Breeding and Genetics, Swedish University of Agricultural Sciences, Uppsala, Sweden). Loan agreement: 331-2017-958.

-Sosnovka 1, Russia. Samples: UR17x27_Rus_568. Key contact: Pavel Kosintsev (Institute of Plant and Animal Ecology, Ural Branch of the Russian Academy of Sciences, Ekaterinburg, Russia).

-Tamiryn Ulaan Khoshuu, Mongolia. Samples: TAM13_Mon_14 (Tomb 13) and TAM22xA2_Mon_5 (Tomb 22). Key contacts: Éric Crubézy (Centre d'Anthropobiologie et de Génomique de Toulouse, CNRS UMR 5288, Université Paul Sabatier, Faculté de Médecine Purpan, 37 Allées Jules Guesde, 31000 Toulouse, France), and Tsagaan Turbat (Archaeological Research Center and Department of Anthropology and Archaeology, National University of Mongolia, Ulaanbaatar, Mongolia).

-Tarquinia monumental complex, Italy. Sample: Tarquinia3206_Ita_275. Key contact: Giovanna Bagnasco Gianni (Dipartimento di Beni Culturali E Ambientali Etruscologia, Universita Degli Studi di Milano, Italy).

-Tominy, Poland. Samples: POZ327_Pol_m5127 (EQ_288, EQ_To6_07; No inv P18/09 (ob. 108), and POZ37_Pol_m5108 (No inv. 55/09; feature 115, layer 163). Key contact: Daniel Makowiecki (Institute of Archaeology, Faculty of History, Nicolaus Copernicus University, Toruń, Poland). Samples are from animal remains excavated (2006 – 2016) by Marcin Szeliga (Institute of Archaeology, Maria Curie-Skłodowska University, Lublin, Poland), and were collected during zooarchaeological research (2018) by Daniel Makowiecki, and stored since at the Institute of Archaeology, Nicolaus Copernicus, Torun.

-Tuse Skole, Denmark. Sample: P135_Den_1806. Key contacts: Kirsten Christensen (museum inspector; Museum West Zealand, Forten 10, 4300 Holbaek, Denmark), and Lone Claudi-Hansen (museum inspector; Museum West Zealand, Forten 10, 4300 Holbaek, Denmark). Sampled with permission from the collection manager at the Collections of the Natural History Museum of Denmark.

-Tyubyak, Russia. Samples: ABA9_Rus_m1786 and ABA10_Rus_m1757. Key contact: Pavel Kosintsev (Institute of Plant and Animal Ecology, Ural Branch of the Russian Academy of Sciences, Ekaterinburg, Russia).

-Ulvehøj, Denmark. Sample: P191_Den_O1663. Key contact: Kristian M. Gregersen (former collection manager of Quaternary Zoology, Natural History Museum of Denmark, Gothersgade 130, 1123 Copenhagen K., Denmark). Sampled with permission from the collection manager at the Collections of the Natural History Museum of Denmark.

-Vejen, Denmark. Sample: P140_Den_O1660. Key contact: Kristian M. Gregersen (former collection manager of Quaternary Zoology, Natural History Museum of Denmark, Gothersgade 130, 1123 Copenhagen K., Denmark). Sampled with permission from the collection manager at the Collections of the Natural History Museum of Denmark.

-Vinkovci, Na-ma, Croatia. Samples: SRKRO009_Cro_m453 and SRKRO011_Cro_331. Key contact: Hrvoje Vulic (Vinkovci Municipal Museum, Trg bana Josipa Šokčevića 16, 32 100 Vinkovci, Croatia). Samples were made available through the Innovation Fund of the Austrian Academy of Sciences (ÖAW) (Grant agreement IF_2015_17).

-Yaloman-II, Russia. Sample: Yal2x24_Rus_m105. Key contact: Alexey A. Tishkin (Department of Archaeology, Ethnography and Museology, Altai State University, Prospekt Lenina, 61, 656049 Barnaul, Russia). Sampled with proper permission and authority by Alexey A. Tishkin from auxiliary collection of the Department of Archaeology, Ethnography and Museology of the Altai State University, under the framework of the Russian Science Foundation project "The world of ancient nomads of Inner Asia: interdisciplinary studies of material culture, sculptures and economy" (No. 22-18-00470).

-Yenikapi, Türkiye. Sample: KSK11b_Tur_829. Key contact: Vedat Onar (Osteoarchaeology Practice and Research Center and Department of Anatomy, Faculty of Veterinary Medicine, Istanbul University-Cerrahpaşa, Istanbul 34320, Turkey).

-Zayukovo 3, Russia. Samples: OSCAE16SP7x1_Rus_604 and OSCAE16SP8_Rus_577. Key contact: Anna Kadieva (State Historical Museum, Department of Archaeological Monuments, Moscow, Red Square 1, Moscow 109012, Russian Federation).

-Zoolongiyn am, Mongolia. Samples: Zoox1_Mon_333 and Zoox2_Mon_271. Key contact: Alexey A. Tishkin (Department of Archaeology, Ethnography and Museology, Altai State University, Prospekt Lenina, 61, 656049 Barnaul, Russia). Sampled with proper permission and authority by Alexey A. Tishkin from auxiliary collection of the Department of Archaeology, Ethnography and Museology of the Altai State University, under the framework of the Russian Science Foundation project "The world of ancient nomads of Inner Asia: interdisciplinary studies of material culture, sculptures and economy" (No. 22-18-00470).

Specimen deposition	Ancient remains were 3D-scanned in our facilities to ensure future morphometric studies, if needed, and stored in our laboratory facilities unless the bone fragment was inevitably destroyed during ancient DNA extraction. Based on the detailed archaeological metadata provided in Table S1, scholars are encouraged to contact Prof. Ludovic Orlando if aim to obtain further sample information.

Dating methods	140 samples were radiocarbon-dated. Their calibrated and uncalibrated dates, 95% confidence intervals, and relevant laboratory codes are provided in Table S1.

☒ Tick this box to confirm that the raw and calibrated dates are available in the paper or in Supplementary Information.

Ethics oversight	*Identify the organization(s) that approved or provided guidance on the study protocol, OR state that no ethical approval or guidance was required and explain why not.*

Note that full information on the approval of the study protocol must also be provided in the manuscript.

Plants

Seed stocks	*Report on the source of all seed stocks or other plant material used. If applicable, state the seed stock centre and catalogue number. If plant specimens were collected from the field, describe the collection location, date and sampling procedures.*
Novel plant genotypes	*Describe the methods by which all novel plant genotypes were produced. This includes those generated by transgenic approaches, gene editing, chemical/radiation-based mutagenesis and hybridization. For transgenic lines, describe the transformation method, the number of independent lines analyzed and the generation upon which experiments were performed. For gene-edited lines, describe the editor used, the endogenous sequence targeted for editing, the targeting guide RNA sequence (if applicable) and how the editor was applied.*
Authentication	*Describe any authentication procedures for each seed stock used or novel genotype generated. Describe any experiments used to assess the effect of a mutation and, where applicable, how potential secondary effects (e.g. second site T-DNA insertions, mosiacism, off-target gene editing) were examined.*
